# Numerical investigation of flow characteristics in the front and rear chambers of centrifugal pump and pump as turbine

**DOI:** 10.1038/s41598-024-62831-4

**Published:** 2024-05-25

**Authors:** Yu-Liang Zhang, Shao-Han Zheng, Yan-Juan Zhao

**Affiliations:** 1 College of Mechanical Engineering, Quzhou University, Quzhou, 324000 China; 2 College of Mechanical Engineering, Zhejiang University of Technology, Hangzhou, 310023 China; 3 College of Information Engineering, Quzhou College of Technology, Quzhou, 324000 China

**Keywords:** Centrifugal pump, Pump as turbine, Pump chamber, Volumetric efficiency, Instability, Energy science and technology, Engineering

## Abstract

To investigate the flow characteristics in front chamber and rear chamber in pump mode and pump as turbine mode, a 3D computational model of a centrifugal pump was established, including the front and rear chamber. Based on Realizable k-ε turbulence model, numerical calculations of incompressible flow were carried out for internal viscous flow in two operating modes. Further analysis was conducted on the flow stability and hydraulic losses under two modes using energy gradient theory and entropy production theory. The numerical simulation results are within reasonable error compared to the experimental results in pump operation mode, which ensures the reliability of the numerical calculation method. The results indicate that the volumetric efficiency in both two modes is on an upward trend with increasing flow, but the volumetric efficiency of the pump mode is more significantly affected by changes in flow; the distribution patterns of dimensionless circumferential velocity and dimensionless radial velocity in the front and rear chambers under two operating modes are similar, but the distribution pattern of dimensionless radial velocity in the front chamber in turbine mode is significantly different from other operating conditions; flow instability is most likely to occur at the outlet of impeller, and the energy loss in clearance of wear-rings is greater than that in the pump chamber.

## Introduction

Centrifugal pump converts mechanical energy into kinetic energy and pressure potential energy of transportation medium, and is widely used in various fields such as petroleum, chemical industry, medicine, power, transportation, metallurgy, agricultural irrigation, etc.^1–4^. Pump as turbine is a type of liquid residual pressure energy recovery equipment, which is widely used for energy utilization in agricultural irrigation^5^, hydroelectric power generation^6^ and urban water supply systems^7^ due to its advantages of simple structure, easy maintenance, and low cost. Improving the performance of centrifugal pump and pump as turbine to achieve high efficiency has been the main goal of pump researchers. Nowadays, numerical simulation methods based CFD provide a powerful and effective means for pump design and optimization, which can not only provide an in-depth analysis of the internal flow, material and energy transfer in the pump flow field, but also provide engineers with quantitative data and visualization results to support decision-making and improvement efforts^8–10^.

In recent years, extensive research has been conducted on centrifugal pumps, revealing that variations in flow within the pump chamber can have a significant impact on its performance and internal flow characteristics. A deeper understanding of this flow characteristic can provide a theoretical basis for optimizing pump chamber structure and improving pump performance^11^. Black et al.^12^ theoretically and experimentally investigated the fluid excitation forces at the sealing ring gap of a centrifugal pump, and explored their effects on the vibration of the pump rotor. Li et al.^13^ proposed a new radial embedded seal (RES) with less fluid leakage and flow-induced force, and significantly reduced tangential force at high rotational speeds. Wu et al.^14^ found that the axial distribution of the flow field in the front pump chamber is asymmetric, with a large vorticity at the inlet of the impeller and a large energy dissipation zone at the outlet. Zeng et al.^15^ deduced the mathematical model of the pump chamber pressure distribution and balance chamber pressure under the design condition and conducted experimental tests to prove that the mathematical model proposed by the authors has high accuracy and universality, which can calculate the pressure distribution in the balance chamber and pump chambers more accurately, and provides a theoretical basis for the study of impeller shroud force. Jin et al.^16^ conducted numerical calculations on the entire flow field of the molten salt pump and analyzed the velocity distribution and energy loss inside the front and rear chambers of a pump, found that the proportion of loss in the front and rear cover plate pump chambers to the total energy consumption and the ratio of loss energy decreased with increasing flow. Dong et al.^17^ found that the change in the diameter of the balance hole has a significant impact on the flow characteristics and disk friction loss in the rear chamber of a centrifugal pump under the rated operating condition. It is also found that the pressure distribution in rear chamber is affected by the shape of the volute, and the difference in different angles is significant^18^. The pressure of the pump chamber near the hub area has a tendency to decrease with the decrease of the axial width of the pump chamber, and when the axial width increases, the pressure at the same radius also increases, and the radial differential pressure decreases^19^. Dong et al. ^20^ used particle model and heterogeneous model to numerically simulate the flow field containing particles in a semi-open impeller centrifugal pump, and studied the pressure distribution characteristics and axial force variation law of the pump chamber containing particle flow centrifugal pump. Wang et al. ^21^ conducted a three-dimensional numerical simulation of 100HZ165-250 centrifugal pump with different widths by using compact local mesh and regional computation techniques to research the influence of the back blades on the pressure distribution in pump chamber, and proposed that there are optimal values of the number and width of the back blades for balancing the axial force. Liu et al.^22^ explored the number of balance holes, diameter and other parameters on the rear pump chamber pressure and axial force, found that when the number of balance holes and the number of vanes is the same, the more the balance of the axial force; balance holes in a certain range of the diameter increases, the pressure at the same location in the pump chamber will subsequently decrease. Zhang et al.^23^ conducted a numerical analysis on the radial distribution of fluid axial velocity in center of the pump chamber at different angles, as well as the radial distribution of fluid pressure in the pump chamber from different angles. The influence of the pump chamber on the hydraulic performance of mixed flow pumps was also studied^24^. Li et al. ^25,26^ investigated the pressure fluctuations and interstage differences existing between the stages of a multistage PAT through experiments and numerical simulations.

In summary, existing literature focuses on studying the flow characteristics of the front and rear chambers in pump operating mode, while the front and rear chambers under pump operating in turbine mode have not been extensively researched yet. This paper carries out numerical calculations on centrifugal pump mode and pump as turbine mode by CFD method, obtains the flow field characteristics in the front and rear chambers and the external characteristics in two operating modes, and further analysis the flow stability and hydraulic loss in the two modes with the help of the energy gradient theory and entropy production theory.

## Calculation model and method

### Calculation model

**Figure 1:**
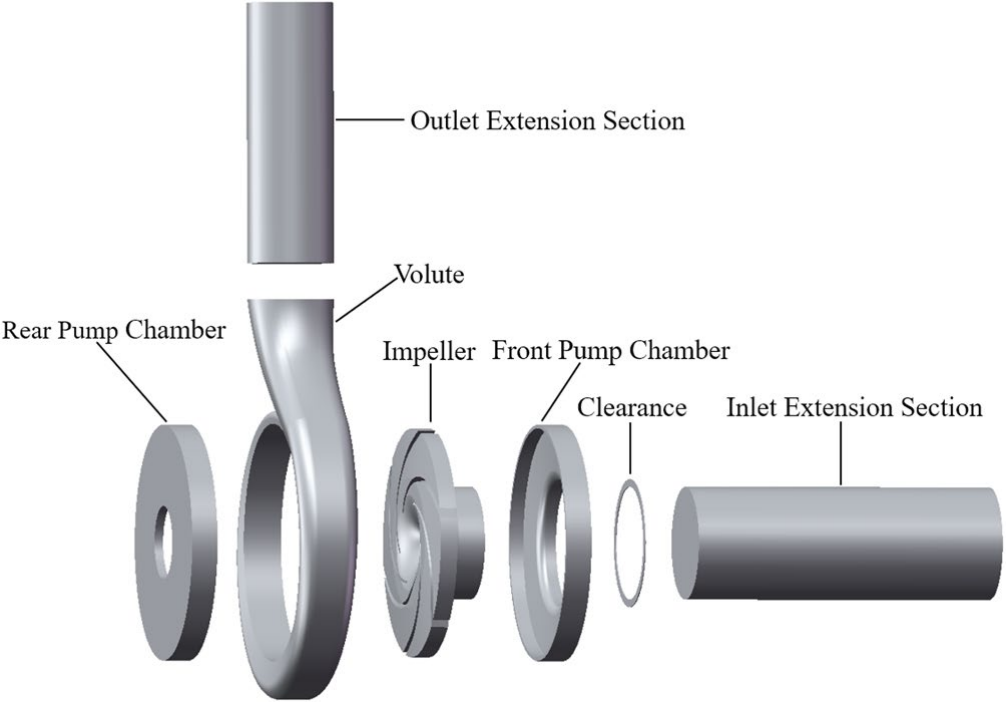
3D calculation domain of centrifugal pump.

**Figure 2:**
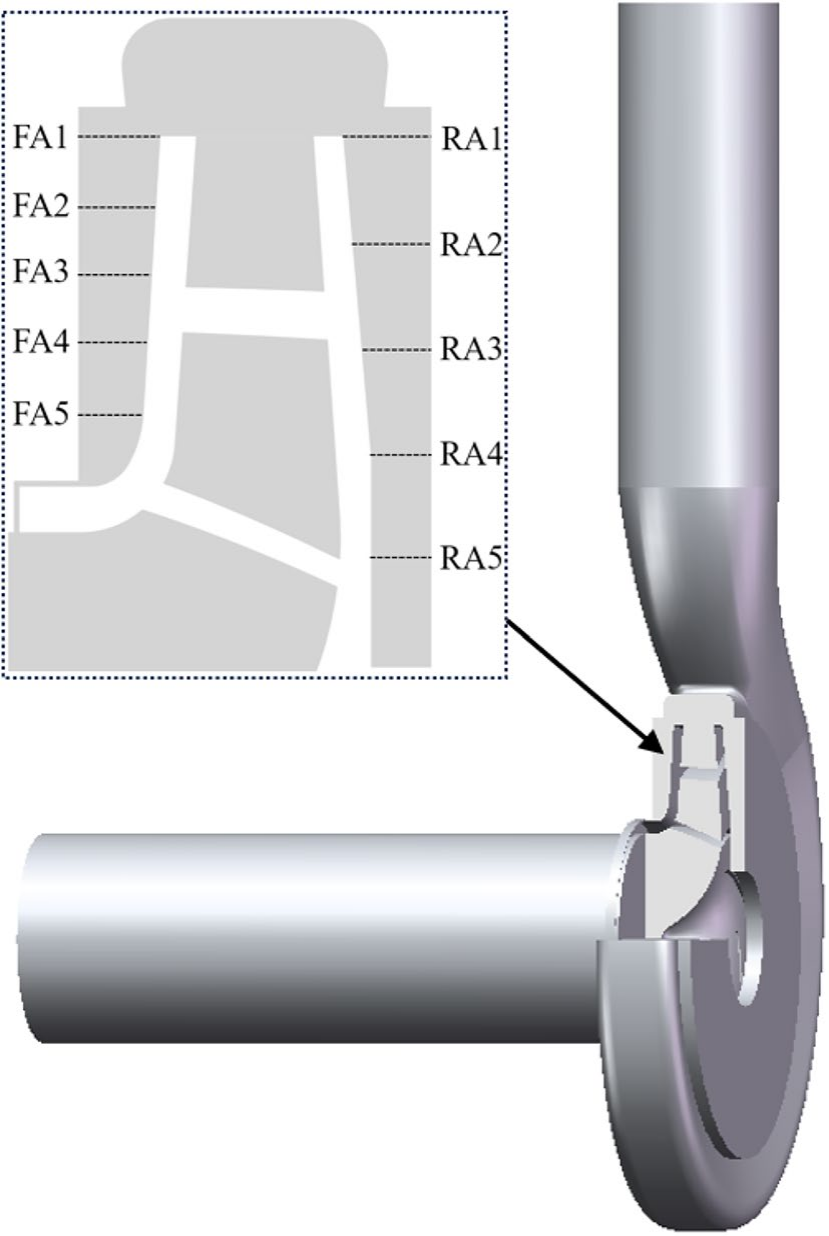
Monitoring position.

This paper takes a single-stage single-suction centrifugal pump model as the research object, the main parameters are: rated flow *Q* = 25 m^3^/h, rated head *H* = 21 m, rated speed *n* = 2900 r/min, blade number *z* = 6, blade wrap angle *φ* = 114°, impeller inlet diameter *D*_1_ = 65 mm, impeller outlet diameter *D*_2_ = 132 mm, impeller outlet width *b* = 10 mm, pump inlet diameter *D*_in_ = 65 mm, pump outlet diameter* D*_out_ = 50 mm. The centrifugal pump three-dimensional full flow channel schematic shown in Fig. 1, including inlet extension section, outlet extension section, volute, impeller, front pump chamber, rear pump chamber, clearance of wear-ring and other flow channel main structure. The conveying medium is clear water at room temperature, with a density of 998.2 kg/m^3^. The ten monitoring positions are located at the 0° axial section of the centrifugal pump near the volute tongue, as shown in Fig. 2. FA1, FA2, FA3, FA4, and FA5 are at *r* = 66 mm, *r* = 60 mm, *r* = 54 mm, *r* = 48 mm, and *r* = 42 mm of the front chamber, respectively; RA1, RA2, RA3, RA4, and RA5 are at *r* = 66 mm, *r* = 57 mm of the rear chamber, respectively, *r* = 48 mm, *r* = 39 mm, *r* = 30 mm at the rear chamber, for which the data were processed respectively.

### Grid independent verification

**Figure 3:**
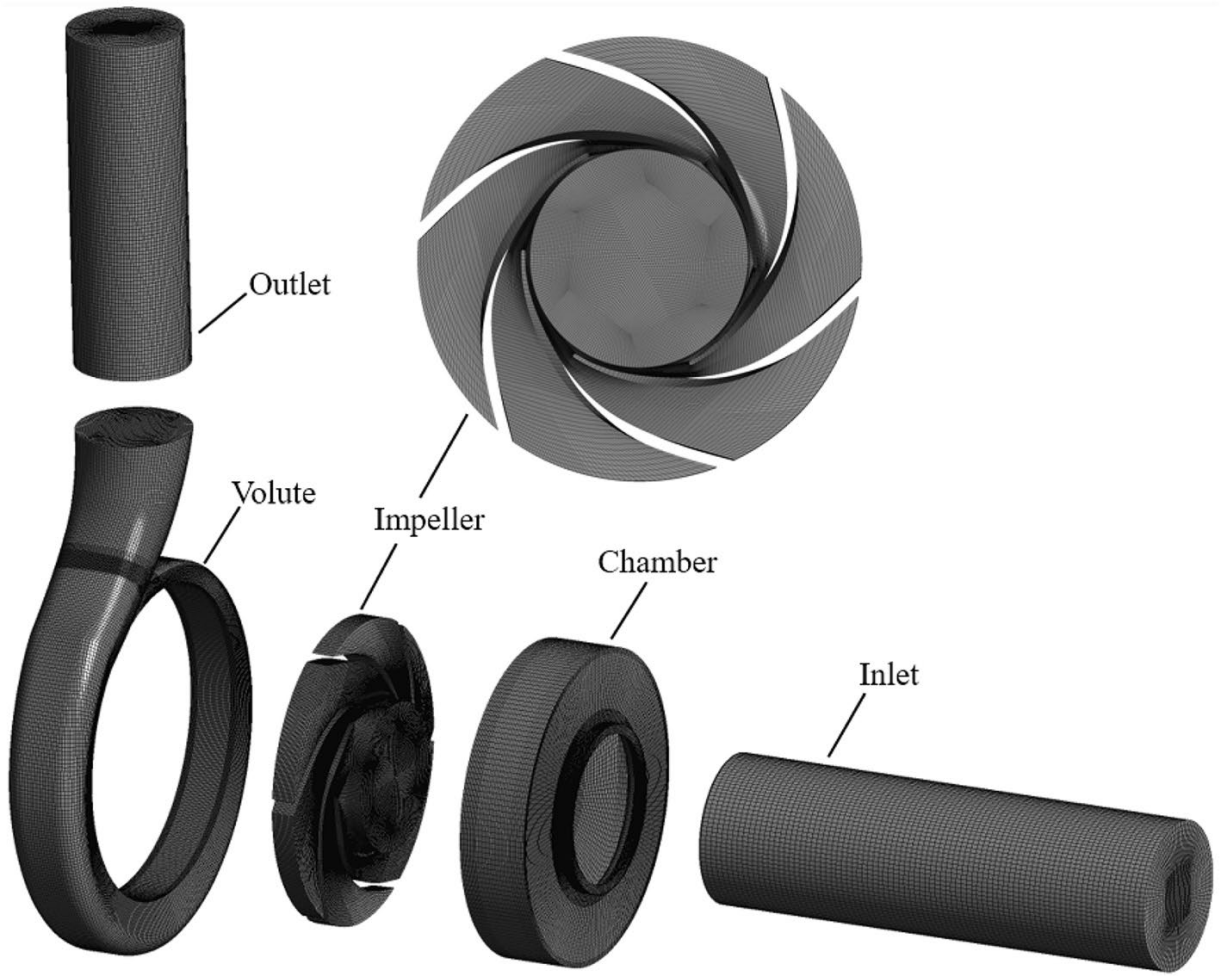
Centrifugal pump grid.

**Figure 4:**
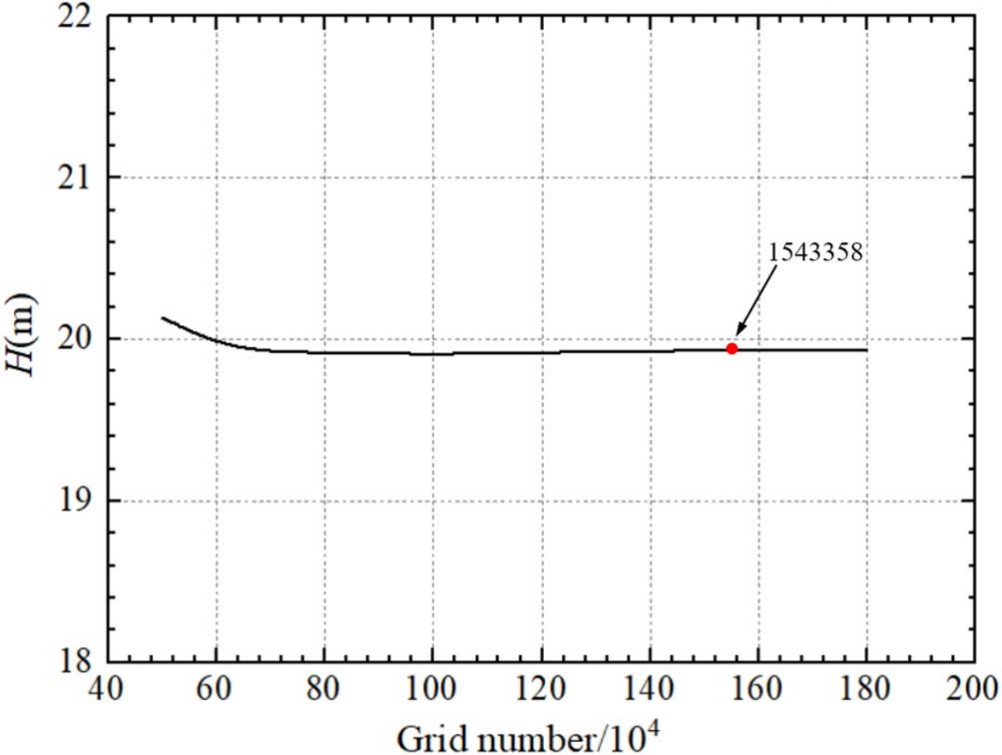
Grid independence verification.

ANSYS-ICEM is used to perform finite element meshing of the centrifugal pump model, and hexahedral structured grid is used for the whole pump model. Appropriate extensions were made at the inlet and outlet of the centrifugal pump, and the grid division is shown in Fig. 3. Figure 4 shows the grid independence verification for different grids number under rated operating condition. It was found that the pump head is basically unchanged when the grids number is greater than 1,543,358. Theoretically, the grids number is directly proportional to the calculation accuracy. Considering computational efficiency, the final number of grids in this paper for 1,543,358.

### Governing equation

Assuming that the fluid motion is incompressible flow, the continuity equation can be simplified as:

1 $$ \frac{{\partial u_{{\text{i}}} }}{{\partial x_{{\text{i}}} }} = 0 $$

The momentum equation (N-S equation) is:

2 $$ \begin{aligned} \frac{{\partial \left( {\rho u_{{\text{j}}} } \right)}}{\partial t} + \frac{{\partial \left( {\rho u_{{\text{i}}} u_{{\text{j}}} } \right)}}{{\partial x_{{\text{j}}} }} & = F_{{\text{i}}} - \frac{\partial \rho }{{\partial x_{{\text{i}}} }} + \frac{\partial }{{\partial x_{{\text{i}}} }}\left[ {\left( {\mu + \mu_{{\text{t}}} } \right)\left( {\frac{{\partial u_{{\text{i}}} }}{{\partial x_{{\text{j}}} }} + \frac{{\partial u_{{\text{j}}} }}{{\partial u_{{\text{i}}} }} - \frac{2}{3}\frac{{\partial u_{{\text{k}}} }}{{\partial x_{{\text{k}}} }}\delta_{{{\text{ij}}}} } \right)} \right] \\ \rho & = \rho_{{\text{v}}} \alpha_{{\text{v}}} + \rho_{1} \left( {1 - \alpha_{{\text{l}}} } \right) \\ \end{aligned} $$

Among them, $u_{{\text{i}}}$, $u_{{\text{j}}}$ are the partial velocities of small volume elements in the medium in all directions; $\rho$ is the density of the medium; $\mu$ and $\mu_{{\text{t}}}$ are the dynamic viscosity and turbulent viscosity of the mixture; $\alpha_{{\text{v}}}$ and $\alpha_{{\text{l}}}$ are the volume fractions of the gas and liquid phases; $\delta_{{{\text{ij}}}}$ is the Kronecker number; $F_{{\text{i}}}$ is the physical force acting on a small volume element of the medium in all directions, and in this article, the physical force is only gravity and is downward along the Z-axis, then, $F_{{\text{x}}} = 0$, $F_{{\text{y}}} = 0$, $F_{{\text{z}}} = \rho {\text{g}}$.

Realizable k-ε turbulence model is chosen, and the turbulent kinetic energy and turbulent kinetic energy dissipation equations are^27^:

3 $$ \frac{\partial (\rho k)}{{\partial t}} + \frac{{\partial \left( {\rho ku_{{\text{i}}} } \right)}}{{\partial x_{{\text{i}}} }} = \frac{\partial }{{\partial x_{{\text{j}}} }}\left[ {\left( {\mu + \frac{{\mu_{{\text{t}}} }}{{\sigma_{{\text{k}}} }}} \right)\frac{\partial k}{{\partial x_{{\text{j}}} }}} \right] + G_{{\text{k}}} - \rho \varepsilon $$

4 $$ \frac{\partial (\rho \varepsilon )}{{\partial t}} + \frac{{\partial \left( {\rho \varepsilon u_{{\text{i}}} } \right)}}{{\partial x_{{\text{i}}} }} = \frac{\partial }{{\partial x_{{\text{j}}} }}\left[ {\left( {\mu + \frac{{\mu_{{\text{t}}} }}{{\sigma_{\varepsilon } }}} \right)\frac{\partial \varepsilon }{{\partial x_{{\text{j}}} }}} \right] + \rho C_{1} E\varepsilon - \rho C_{2} \frac{{\varepsilon^{2} }}{{k + \sqrt {v\varepsilon } }} $$

where $$C_{1} = \max \left( {0.43,\;\frac{\eta }{\eta + 5}} \right)$$, $$\eta = \left( {2E_{{{ij}}}^{2} } \right)^{1/2} \frac{k}{\varepsilon }$$, $$E_{{{ij}}} = \frac{1}{2}\left( {\frac{{\partial u_{{i}} }}{{\partial x_{{j}} }} + \frac{{\partial u_{{j}} }}{{\partial x_{{i}} }}} \right)$$, $$C_{\mu } = \frac{1}{{A_{0} + A_{{s}} U^{ * } k/\varepsilon }}$$, $$A_{0} = 4$$, $$A_{{s}} = \sqrt 6 \cos \phi$$, $$\sigma_{{k}} = 1$$, $$\sigma_{\varepsilon } = 1.2$$, $$C_{2} = 1.9$$, $$U^{ * } = \sqrt {E_{{{ij}}}^{2} +\Omega _{{{ij}}} }$$, $$\Omega _{{{ij}}} = {\overline{\Omega }}_{{{ij}}} - \varepsilon_{{{ijj}}} \omega .$$

### Boundary conditions

In this paper, the model is calculated based on the FLUENT module in ANSYS software and Realizable k-ε turbulence model. The SIMPLE algorithm is used for the coupling calculation of pressure and velocity. Clear water medium is selected for calculation, and the flow mode inside the pump is incompressible flow.

In pump mode, the inlet is set as a velocity inlet, assuming that the inlet velocity is uniformly distributed along the axial direction, imposed with 5% turbulence intensity and 10 turbulent viscosity ratio; the outlet is set as outflow, which has no effect on upstream. In turbine mode, the inlet is the outlet of the pump mode and is set as velocity inlet, imposed with 5% turbulence intensity and 10 turbulent viscosity ratio; the outlet is the inlet of the pump mode and is set as outflow. The wall surface is a non-slip smooth wall, and a standard wall function is used in the near-wall region.

The pressure secondary relaxation term adopts a second-order scheme, the momentum secondary relaxation term adopts a second-order upwind scheme, and the turbulent kinetic energy secondary relaxation term and turbulent dissipation rate term both adopt a first-order upwind scheme. The under-relaxation coefficients for the pressure–velocity coupling equation, the momentum equation, and the equations for the turbulent kinetic energy and its dissipation are 0.3, 0.7, 0.8, and 0.8, respectively. The convergence criterion is 1 × 10^−5^ for the residuals of those equations^28^.

## Result analysis

### Verification of calculating method

**Figure 5:**
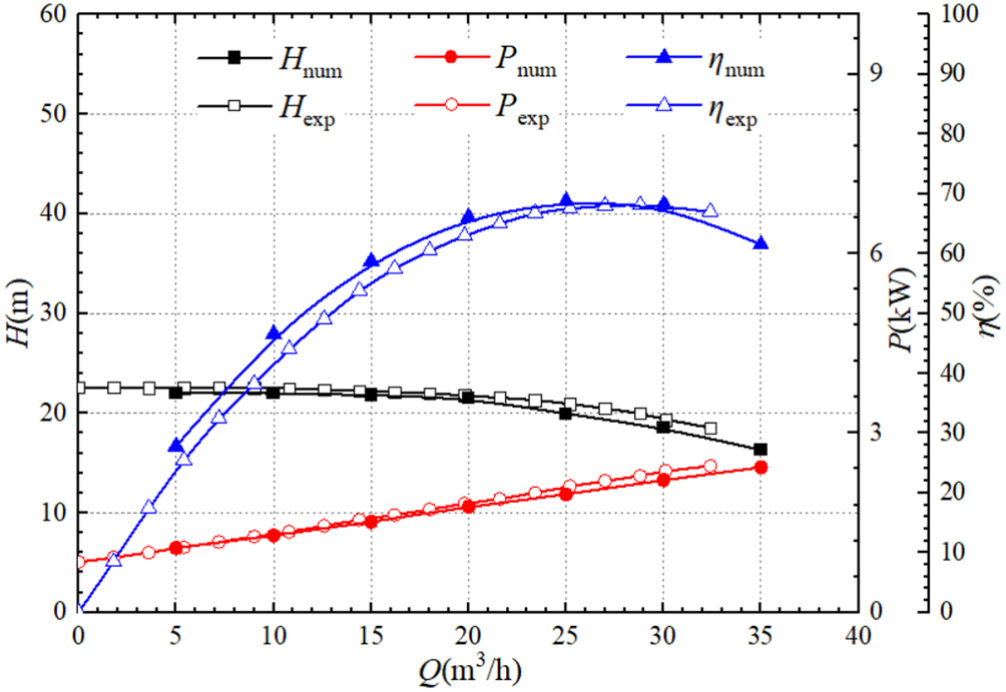
Centrifugal pump external hydraulic performance.

Figure 5 shows the comparison of the performance calculation curve and experimental curve of the centrifugal pump studied in this article. At rated flow of 25 m^3^/h, the experimental head is 21.0 m, the calculated head is 19.97 m, and the relative error is 4.86%; The experimental shaft power is 2.11 kW, the calculated shaft power is 1.97 kW, and the relative error is 6.64%; The experimental efficiency is 67.5%, the computational efficiency is 68.77%, and the relative error is 1.88%. In the flow of 15 m^3^/h, the error of the calculated value of head relative to the experimental value is 2.07%, the error of the calculated value of shaft power relative to the experimental value is 2.17%, and the error of the calculated value of efficiency relative to the experimental value is 5.49%; In the flow of 20 m^3^/h, the error of the calculated value of head relative to the experimental value is 1.33%, the error of the calculated value of shaft power relative to the experimental value is 2.74%, the efficiency of the calculated value of the error relative to the experimental value of 4.93%; In the flow of 30 m^3^/h, the error of the calculated value of head relative to the experimental value is 4.32%, the error of the calculated value of shaft power relative to the experimental value is 6.46%, and the error of the calculated value of efficiency relative to the experimental value is 0.51.%. According to calculations, the maximum errors in head and shaft power both occur in the working condition of 25 m^3^/h; The maximum error in calculating efficiency relative to experimental efficiency occurs in the working condition of 10 m^3^/h, which is 5.78%. The overall calculation results are slightly higher than the experimental results, and the reason for this phenomenon is that the roughness of the calculated model wall is lower than the actual model, but the errors are within the allowable range, indicating that the numerical calculations used in this paper are reliable and the results obtained are reliable. In the next step of numerical calculation, the method will be used to complete the series of calculation model in pump mode and turbine mode.

### External characteristics comparison

**Figure 6:**
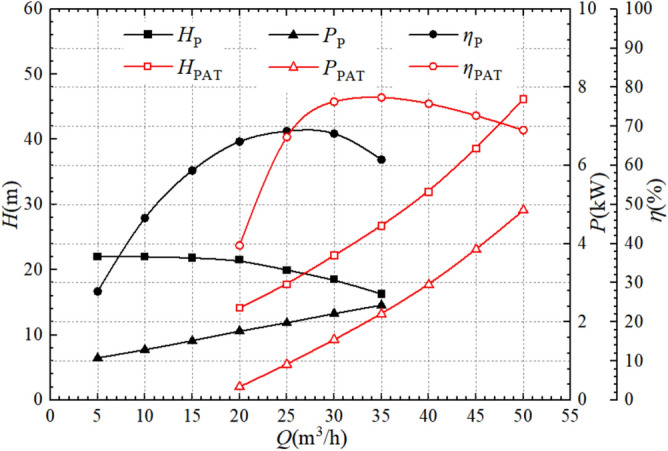
External hydraulic performance comparison of two operating modes.

Figure 6 shows the comparison of the external characteristics in pump mode and turbine mode. The efficiency curves of two modes have a similar variation pattern, the efficiency of each first increases and then decreases with the increase of the flow. The maximum pump efficiency is 68.77%, the maximum turbine efficiency is 77.39%, the highest efficiency ratio of 1:1.13, the most efficient flow condition for the two operating modes is the rated working conditions. The maximum efficiency increases by 12.53% when the pump is reversed for the turbine level. The efficiency of two modes is basically equal when the flow is 25 m^3^/h, which is the optimal flow for the pump, but lower than the optimal flow for the turbine. As shown in the figure, the efficiency of the pump and the turbine varies significantly at low operating flows. The efficiency change rates of the pump at flows of 10 m^3^/h, 15 m^3^/h, and 20 m^3^/h are 40.25%, 20.73%, and 11.19%, respectively. The efficiency at each point is 46.54%, 58.71%, and 66.11%. When the flows are 25 m^3^/h and 30 m^3^/h, the efficiency change rates of the pump as turbine are 70.00% and 11.84%, respectively, and the efficiency at each point are 67.27% and 76.31%. It can be concluded that under conditions below the optimal flow, the efficiency increase of two modes continuously decreases as the flow increases. When the flows are 25 m^3^/h and 30 m^3^/h, the efficiency change rate of the pump are 4.03% and 0.91%, and the efficiency at each point are 68.77% and 68.14%, respectively. When the flows are 35 m^3^/h, 40 m^3^/h, and 45 m^3^/h, the efficiency change rates of the pump as a turbine are 1.42%, 2.09%, and 4.06%, and the efficiency at each point is 77.39%, 75.81%, and 72.73%, respectively. It comes to a conclusion that the efficiency of the pump and turbine in the high-efficiency zone changes relatively little.

The difference in head between pump and turbine with flow variation is significant. The pump head decreases continuously as the flow increases, while the head of the turbine increases continuously. The pump head and turbine head under optimal flow conditions are 19.97 m and 26.68 m respectively, with a ratio of 1:1.34. The head of the pump increased by 6.75 m during the reversal process. At 27 m^3^/h, the two heads are basically equal, about 19.36 m. With the flow rising from 5 to 20 m^3^/h, the pump head decreases by 0.53 m, and with the flow rising from 20 to 35 m^3^/h, the pump head decreases by 5.22 m. But the turbine head rises continuously as the flow increases, and with the flow rising from 20 to 50 m^3^/h, the turbine head increases by 32.01 m. It can be concluded that changes in flow have a greater impact on turbine head. When the flows are 10 m^3^/h, 15 m^3^/h, 20 m^3^/h, 25 m^3^/h, 30 m^3^/h, and 35 m^3^/h, the pump heads are 22.03 m, 21.82 m, 21.51 m, 19.97 m, 18.51 m, and 16.33 m, respectively. The head change rates at each point are − 0.01%, − 0.95%, − 1.42%, − 7.16%, − 7.31%, and − 11.78%, which can be seen that the decrease rate in pump head gradually increases as the flow increases. When the flows are 25 m^3^/h, 30 m^3^/h, 35 m^3^/h, 40 m^3^/h, 45 m^3^/h, and 50 m^3^/h, the head of the pump as a turbine are 17.76 m, 22.20 m, 26.68 m, 31.91 m, 38.60 m, and 46.16 m, respectively. The head change rates at each point are 25.51%, 25.00%, 20.18%, 19.60%, 20.97%, and 19.59%. It can be found that the rate of change of the turbine head is greater than that of the pump.

The pump shaft power and the turbine shaft power have similar patterns of change. The pump shaft power and the turbine shaft power under the optimal flow condition is 1.97 W and 2.20 kW respectively, with a ratio of 1:1.12. The shaft power of the turbine is 0.23 kW higher than that of the pump. At 35 m^3^/h, the two shaft powers are closest to each other. The pump shaft power increases by 1.35 kW with the flow rising from 5 to 35 m^3^/h, and the shaft power of the pump as turbine increases by 4.51 kW with the flow rising from 20 to 50 m^3^/h. When the flows of the pump are 10 m^3^/h, 15 m^3^/h, 20 m^3^/h, 25 m^3^/h, 30 m^3^/h, and 35 m^3^/h, the shaft power is 1.29 kW, 1.52 kW, 1.77 kW, 1.97 kW, 2.22 kW, and 2.43 kW, respectively. The power change rates at each point are 16.28%, 15.13%, 16.45%, 11.30%, 12.69%, and 9.46%. When the pump as turbine at flows of 25 m^3^/h, 30 m^3^/h, 35 m^3^/h, 40 m^3^/h, 45 m^3^/h, and 50 m^3^/h, the shaft power is 0.91 kW, 1.55 kW, 2.20 kW, 2.94 kW, 3.85 kW, and 4.85 kW, respectively. The power change rates at each point are 167.65%, 70.33%, 41.94%, 33.64%, 30.95%, and 25.97%. It is evident that as the flow increases, the rate of increase in turbine power is greater, and the turbine power is greatly affected by changes in flow.

**Figure 7:**
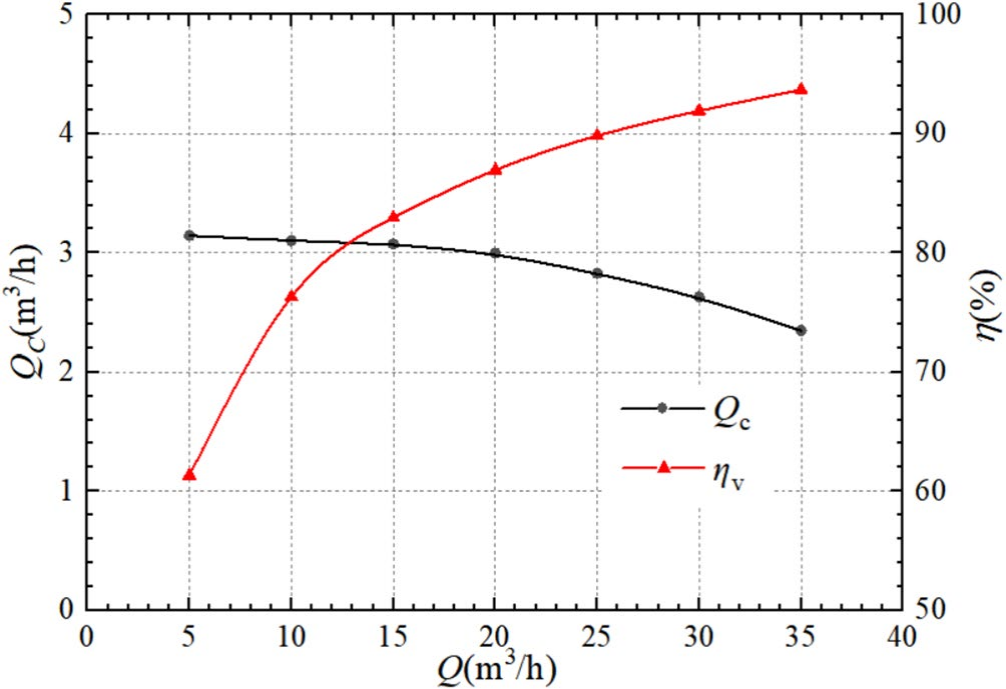
Leakage and volumetric efficiency of centrifugal pump.

Figure 7 shows the relationship between flow leakage at the centrifugal pump wear ring clearance and volumetric efficiency with the operating flow variation. The leakage flow gradually decreasing with the increase in flow, while the volumetric efficiency shows a gradually increasing trend. At low flows, the volumetric efficiency increases rapidly. At the working condition flow of 5 m^3^/h, the leakage is 3.14 m^3^/h, and the volumetric efficiency is 61.34%; at the rated working condition, the leakage is 2.83 m^3^/h, and the volumetric efficiency is 89.83%; at the maximum calculated flow of 35 m^3^/h, the leakage is 2.35 m^3^/h, and the volumetric efficiency is 93.70%. It is evident that with the increase of working condition flow, the rate of increase of volumetric efficiency shows a trend of gradual decrease. This is because as the flow rate increases, the overall pressure inside the pump gradually decreases, and the pressure difference between the front and rear chambers and the impeller outlet also gradually decreases, resulting in a decrease of the fluid leakage from the impeller outlet to the pump chamber. Due to this leakage being detrimental to the efficiency of the system, the smaller the leakage, the greater the volumetric efficiency of the pump.

**Figure 8:**
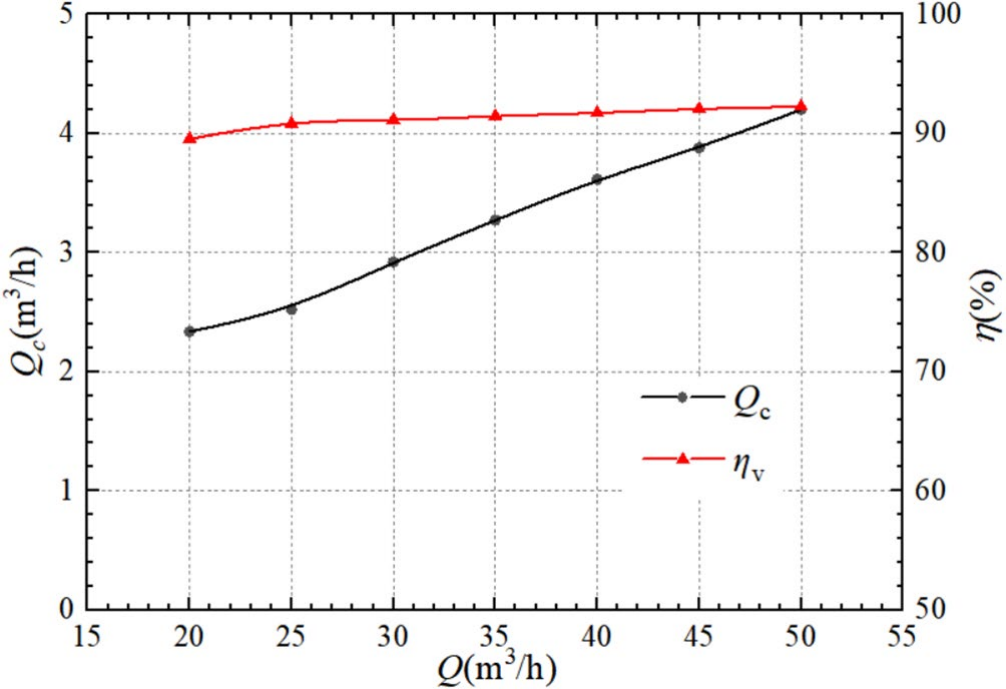
Leakage and volumetric efficiency of pump as turbine.

Figure 8 shows the relationship between flow leakage at the wear ring clearance and volumetric efficiency with the operating flow variation when the pump is working as a turbine. Unlike Fig. 7, as the operating flow increases, the leakage at the clearance and volumetric efficiency of the turbine show a gradually increasing trend. At 20 m^3^/h, the leakage rate is 2.34 m^3^/h, and the volumetric efficiency is 89.53%; At the rated working condition of 35 m^3^/h, the leakage rate is 3.27 m^3^/h, and the volumetric efficiency is 91.44%; At the maximum calculated flow of 50 m^3^/h, the leakage rate is 4.20 m^3^/h, and the volumetric efficiency is 92.24%. It can be found that as the flow increases, the leakage rate at the clearance increases significantly, while the volumetric efficiency is less affected by flow and the growth rate is relatively slow.

### Front and rear chamber velocity analysis in pump mode

**Figure 9:**
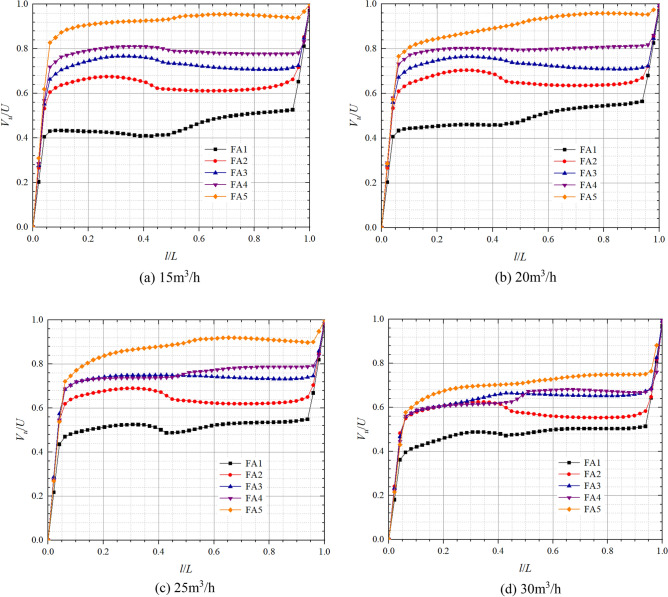
Dimensionless circumferential velocity axial distribution in the front chamber at pump mode.

**Figure 10:**
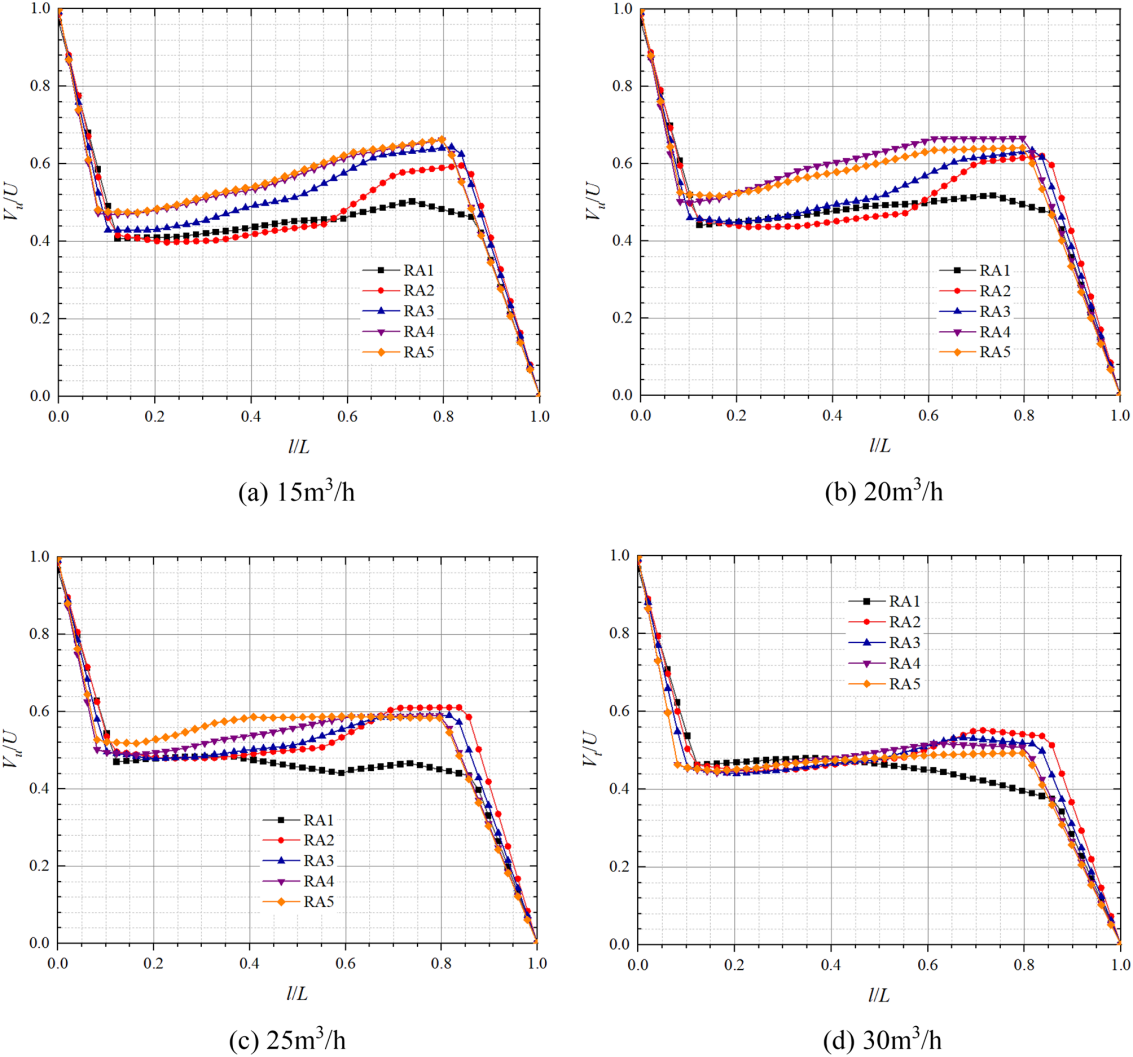
Dimensionless circumferential velocity axial distribution in the rear chamber at pump mode.

Figures 9 and 10 show the axial distribution of dimensionless circumferential velocities in the front pump chamber and rear pump chamber respectively in pump mode. *l*/*L* is the axial relative length; *V*_*u*_/*U* is the dimensionless circular velocity, where *V*_*u*_ is the fluid circumferential velocity at different positions in axial direction, and *U* is the rotational velocity at the corresponding position, *U* = *ωr*. From the figure below, it can be found that the flow in the pump chamber can be divided into a boundary layer near the wall and a core flow region^29^. The dimensionless circumferential velocity in boundary layer near the impeller cover plate is relatively large, and the closer it is to the cover plate, the closer its circumferential velocity is to the impeller rotation speed, so the dimensionless circumferential velocity is closer to 1; The circumferential velocity in the boundary layer on the pump wall side is relatively small, so the closer it is to the pump wall, the closer its dimensionless circumferential velocity is to 0.

In the front chamber, the relative positions of the boundary layer in pump mode are about 0–0.05 and 0.96–1, and the relative positions of the core flow region range from 0.05 to 0.96; In the rear chamber, the relative positions of the boundary layer are about 0–0.1 and 0.85–1, and the relative positions of the core flow region range from 0.1 to 0.85. The dimensionless circumferential velocity in boundary layer varies approximately linearly with relative position and more significantly than in the core flow region. The axial variation of the dimensionless circumferential velocity in core flow region is small but large along the radial direction. At 25 m^3^/h, the dimensionless circumferential velocity variations in core flow regions of FA1, FA2, FA3, FA4, and FA5 are in the ranges of 0.44–0.55, 0.62–0.65, 0.68–0.75, 0.69–0.79, 0.72, 0.90 respectively; And those of the core flow regions of RA1, RA2, RA3, RA4, and RA5 are in the ranges of 0.43–0.47, 0.49–0.61, 0.49–0.59, 0.50–0.59, 0.52–0.58 respectively. From the figure and the data, it can be found that the dimensionless circumferential velocity in core flow region of front chamber has a tendency to increase slowly towards the front cover plate end, while the dimensionless circumferential velocity in core flow region of rear chamber has a tendency to increase towards the pump wall side, which is the opposite of the trend in the front chamber. As the radius of the axial section decreases, the circumferential velocities in the core flow region and the induced velocities on this radius are gradually approaching each other, which indicates that the shear stress in the cover increases with increasing radius and the gradient of the velocity change increases. It is noteworthy that the radial difference in dimensionless circumferential velocity in front pump chamber is significantly higher than that in the rear pump chamber. At the flow of 30 m^3^/h, the dimensionless circumferential velocities at FA3, FA4, and FA5 of the front pump chamber are obviously smaller, and the variation ranges are reduced from 0.68 to 0.75, 0.69  to 0.79, 0.72  to 0.90, 0.55  to 0.68, 0.56 to 0.68, 0.57 to 0.76, respectively. The dimensionless circumferential velocities of the rear pump chamber are approximately equal at relative positions 0–0.5, and the dimensionless circumferential velocities in the core flow region increase with increasing radius at relative positions greater than 0.5, while the dimensionless circumferential velocities become minimum at RA1.

**Figure 11:**
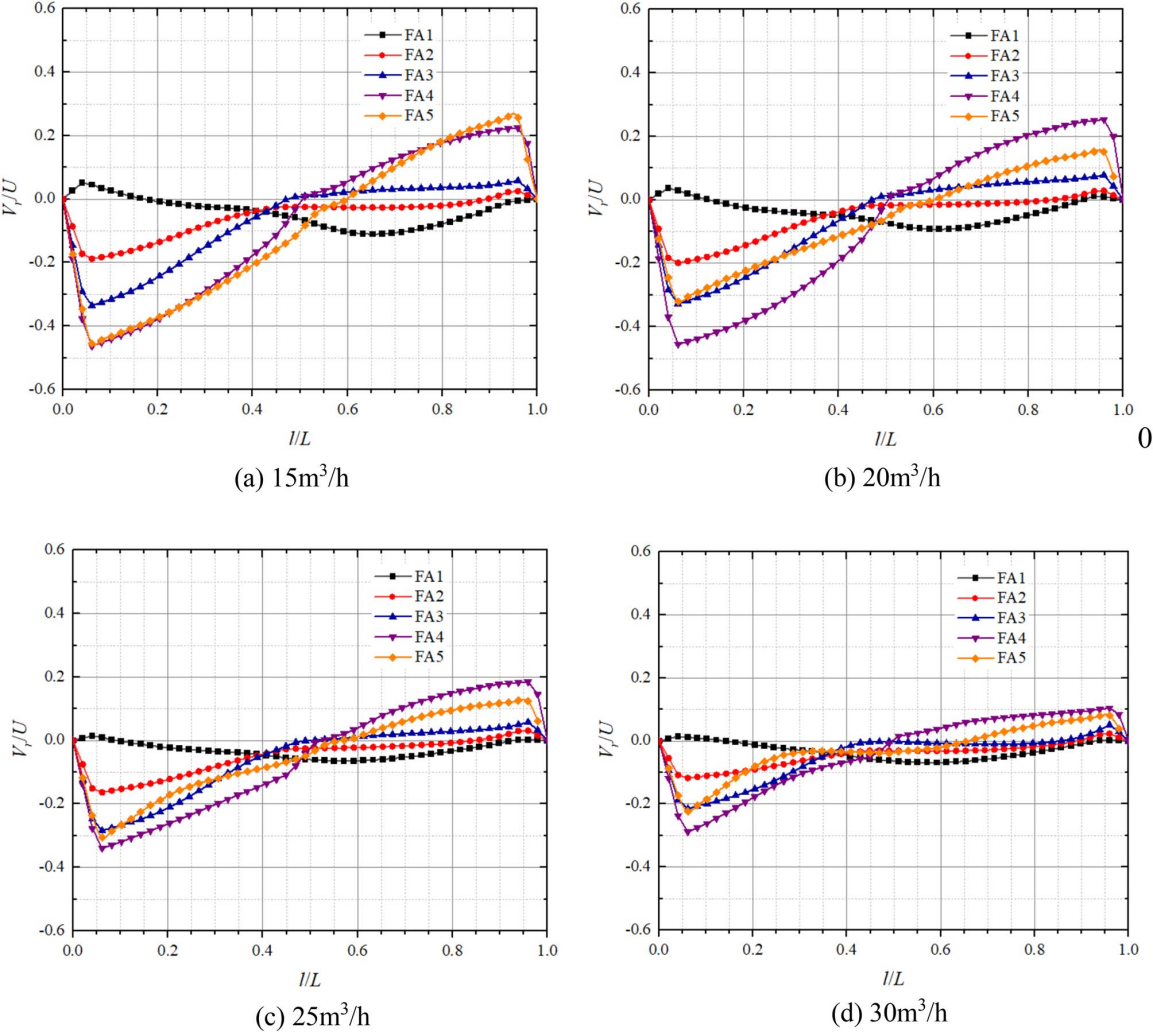
Dimensionless radial velocity axial distribution in the front chamber at pump mode.

**Figure 12:**
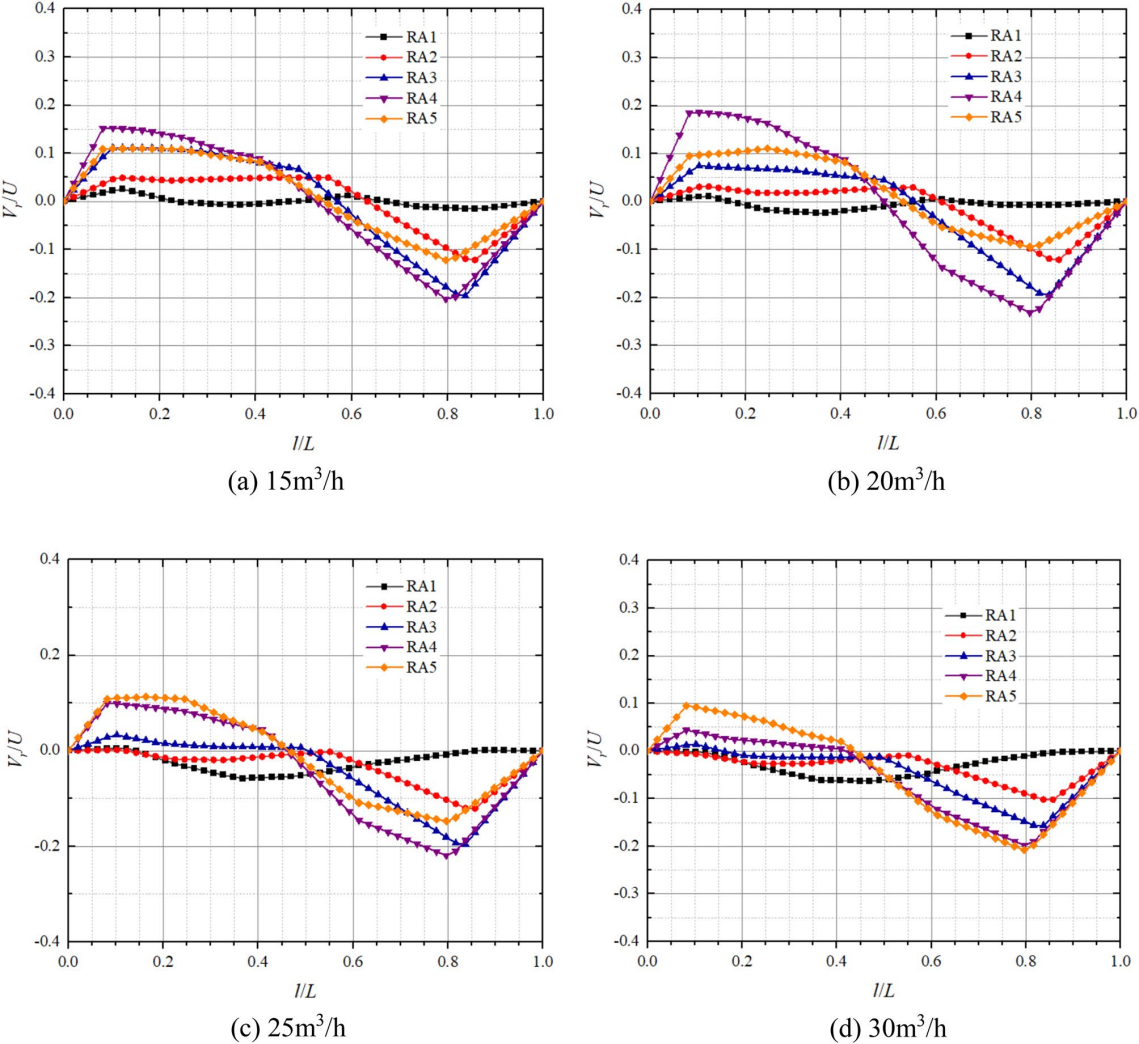
Dimensionless radial velocity axial distribution in the rear chamber at pump mode.

Figures 11 and 12 show the axial distribution of dimensionless radial velocities in the front pump chamber and rear pump chamber respectively in pump mode. *l*/*L* is the axial relative length;* V*_*r*_/*U* is the dimensionless radial velocity, where *V*_*r*_ is the fluid radial velocity at different positions in axial direction, and* U* is the rotational velocity at the corresponding position,* U* = *ωr*. Radial velocity on the impeller front and rear cover plate wall and pump casing wall are all 0. From the graph, it can be observed that the axial variation of dimensionless radial velocity is significantly influenced by the turbulent boundary layer and the core flow zone, both showing a trend of larger on both sides and smaller in the middle. Moreover, the direction of dimensionless radial velocity on the cover plate side and pump shell side is opposite, and the fluid completes the transition of radial velocity direction in the core flow zone. This is because the rotation of the impeller causes a higher circumferential velocity of the fluid near the front and rear cover plates, generating a greater centrifugal force that makes the fluid to flow from the pump chamber to the volute. The circumferential velocity of the fluid near the pump casing is relatively small, making it difficult for centrifugal force to overcome the radial pressure difference, resulting in the radial velocity direction on the pump wall side being opposite to that on the cover plate side.

At the flow of 15 m^3^/h, the maximum dimensionless radial velocities on the side of the pump casing at FA1, FA2, FA3, FA4, FA5 are 0.05, − 0.19, − 0.34, − 0.46, − 0.45 respectively; And on the side of the front cover plate, the maximum dimensionless radial velocities are − 0.01, 0.03, 0.06, 0.22, 0.26 respectively. At the flow of 25 m^3^/h, the maximum dimensionless radial velocities on the pump casing side at FA1, FA2, FA3, FA4, and FA5 are 0.01, − 0.16, − 0.28, − 0.34, and − 0.31, respectively; and the maximum dimensionless radial velocities on the front cover plate side are 0.01, 0.03, 0.06, 0.12, and 0.18, respectively. It can be found that the maximum dimensionless radial velocity is significantly larger on the pump wall side compared to the front cover plate side and tends to decrease on both sides with increasing flow. In particular, the dimensionless radial velocity of the pump casing side at FA5 varies the most with flow from − 0.45 at 15 m^3^/h to − 0.22 at 30 m^3^/h, whereas the dimensionless radial velocities of FA1, FA2, and FA3 on the front cover plate side are close to 0 and vary very little with flow. The dimensionless radial velocities on both sides increase with decreasing radius, but the radial velocity of FA5, which has the smallest radius, is smaller than that of FA4.

In the rear pump chamber, the maximum dimensionless radial velocity is also significantly larger on the pump wall side compared to the rear cover plate side, with larger variations in RA4 and RA5 as the flow increases. At flows of 15 m^3^/h, 20 m^3^/h, 25 m^3^/h, and 30 m^3^/h, the maximum dimensionless radial velocities of the rear cover plate side RA4 are 0.15, 0.18, 0.09, and 0.04, respectively; the maximum dimensionless radial velocities of the rear cover plate RA5 are 0.11, 0.09, 0.11, and 0.09, respectively; the maximum dimensionless radial velocities of the pump casing side RA4 are − 0.20, − 0.23, − 0.22, − 0.19; and the maximum dimensionless radial velocities of pump casing side RA5 are − 0.12, − 0.09, − 0.15, − 0.21, respectively. As the flow and radius increase, the dimensionless radial velocities on both sides of the rear chamber change from increasing to decreasing to gradually increasing. Most notably, the dimensionless radial velocities at FA1 and RA1 are always in a smaller state and are less affected by the flow. It can also be found that the dimensionless radial velocity on the pump wall side is greater than that on the cover side. This is because the rotation of the cover plate causes centrifugal force in the fluid near the cover plate, and there is a radial pressure difference in the pump chamber, resulting in a lower radial velocity on the cover plate side than on the pump wall side.

### Front and rear chamber velocity analysis in turbine mode

**Figure 13:**
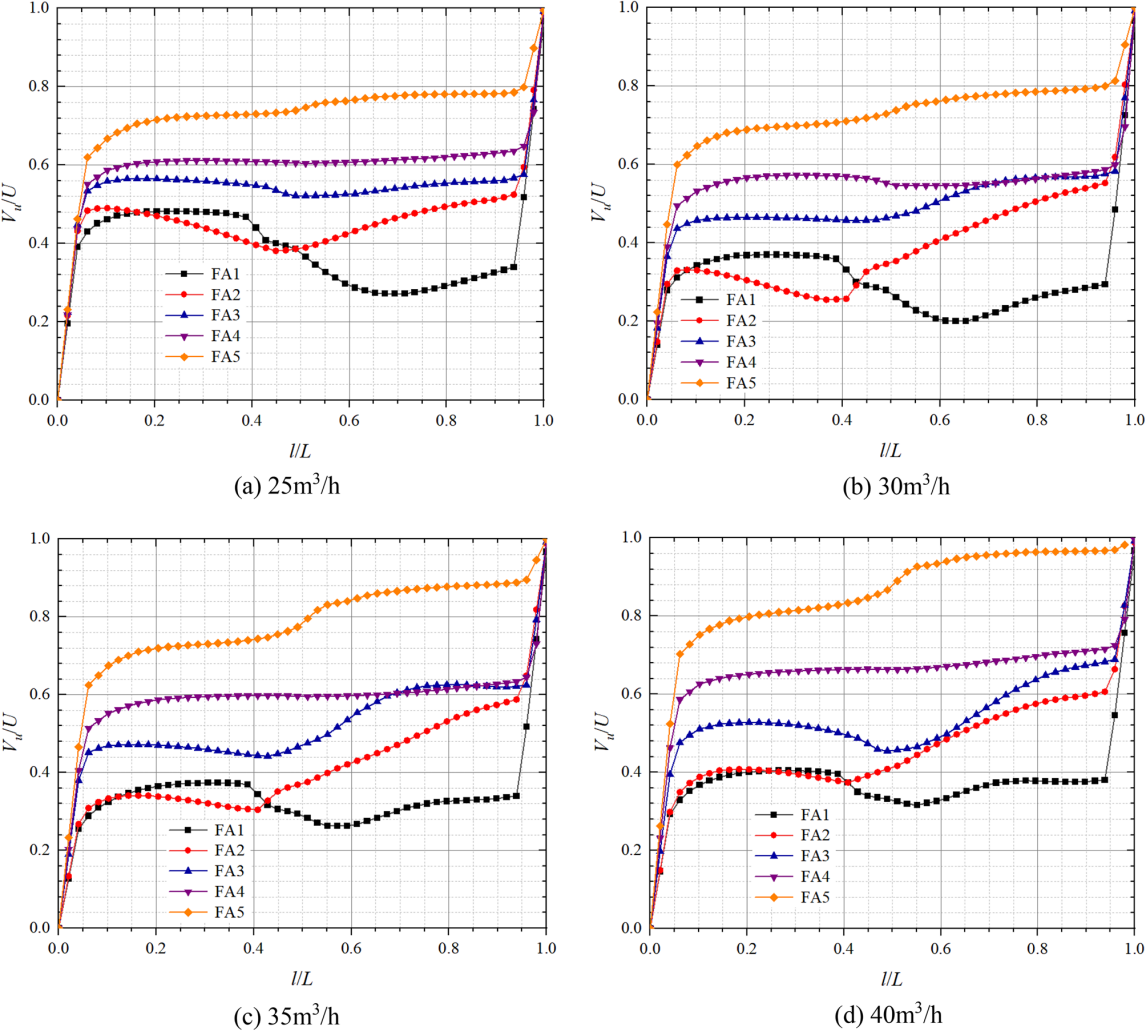
Dimensionless circumferential velocity axial distribution in the front chamber at turbine mode.

**Figure 14:**
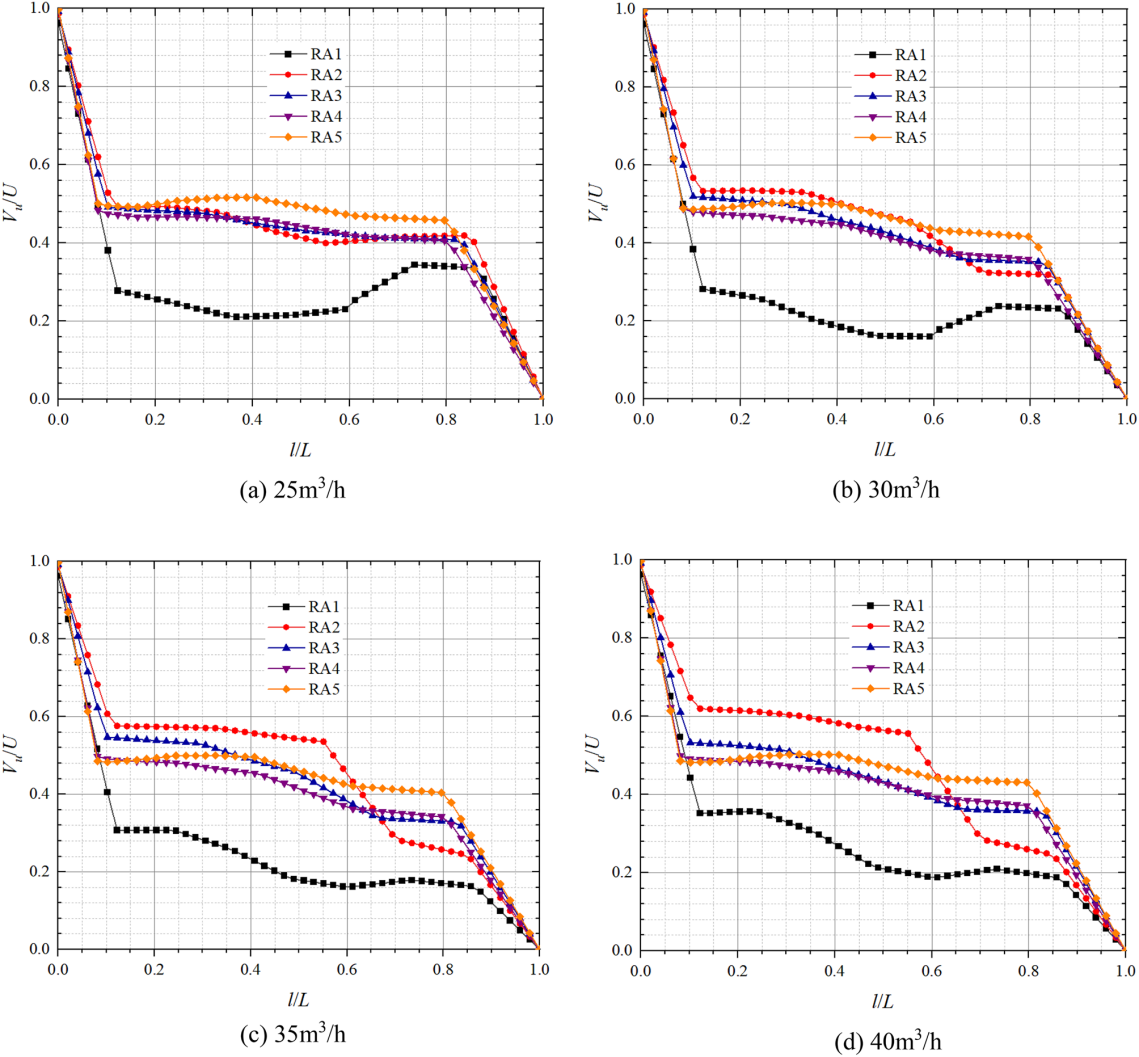
Dimensionless circumferential velocity axial distribution in the rear chamber at turbine mode.

Figures 13 and 14 show the axial distribution of dimensionless circumferential velocities in the front and the rear chambers respectively in turbine mode. The dimensionless circumferential velocity of the front and rear chambers during turbine operation has a similar variation pattern to that of the pump. The dimensionless circumferential velocity varies more significantly along the axial direction in the boundary layer and along the radial direction in the core flow region, and increases with the decrease of radius in the core flow region. In the front chamber, the relative positions of the turbulent boundary layer are 0–0.06 and 0.94–1, and the relative position range of the core flow zone is 0.06–0.94; In the rear chamber, the relative positions of the turbulent boundary layer are 0–0.1 and 0.82–1, and the relative position range of the core flow zone is 0.1–0.82.

From the graph, it can be found that the dimensionless circumferential velocity in the core flow area of the front and rear chambers both show a trend of increasing towards the cover plate side. For the core flow area, at the flow of 25 m^3^/h, the dimensionless circumferential velocity variation ranges of FA1, FA2, FA3, FA4, and FA5 are 0.34–0.43, 0.48–0.52, 0.53–0.58, 0.55–0.65, and 0.62–0.80, respectively; The dimensionless circumferential velocity variation ranges of RA1, RA2, RA3, RA4, and RA5 are 0.28–0.33, 0.40–0.53, 0.39–0.50, 0.41–0.48, and 0.46–0.50, respectively. At the flow of 35 m^3^/h, the dimensionless circumferential velocity variation ranges of FA1, FA2, FA3, FA4, and FA5 are 0.29–0.34, 0.31 ~ 0.59, 0.45–0.62, 0.51–0.64, and 0.62–0.89, respectively; The dimensionless circumferential velocity variation ranges of RA1, RA2, RA3, RA4, and RA5 are 0.16–0.31, 0.25–0.58, 0.33–0.54, 0.33–0.48, and 0.40–0.50, respectively. From this, it can be concluded that in the core flow area, the radial variation of dimensionless circumferential velocity in the front and rear chambers during turbine operation is less affected by flow changes; The radial difference in dimensionless circumferential velocity in the rear chamber is smaller than that in the front chamber, but the dimensionless circumferential velocity at RA1 is significantly smaller than at other positions in the rear chamber.

Specifically, there is a significant axial variation in the dimensionless circumferential velocity at FA1 and FA2 under four different flows. The axial fluctuation in the dimensionless circumferential velocity at FA3 gradually increases with increasing flow. The dimensionless circumferential velocity of FA1 starts to decrease significantly at the relative position of about 0.39 and then rises slowly at the relative position of about 0.6; the dimensionless circumferential velocities of FA2 and FA3 start to increase significantly at relative positions of 0.41 and 0.45, respectively. As the flow increases, the dimensionless circumferential velocity fluctuations at RA2 in the rear chamber become gradually more pronounced and start to become significantly smaller at the relative position of about 0.55 to 0.7.

**Figure 15:**
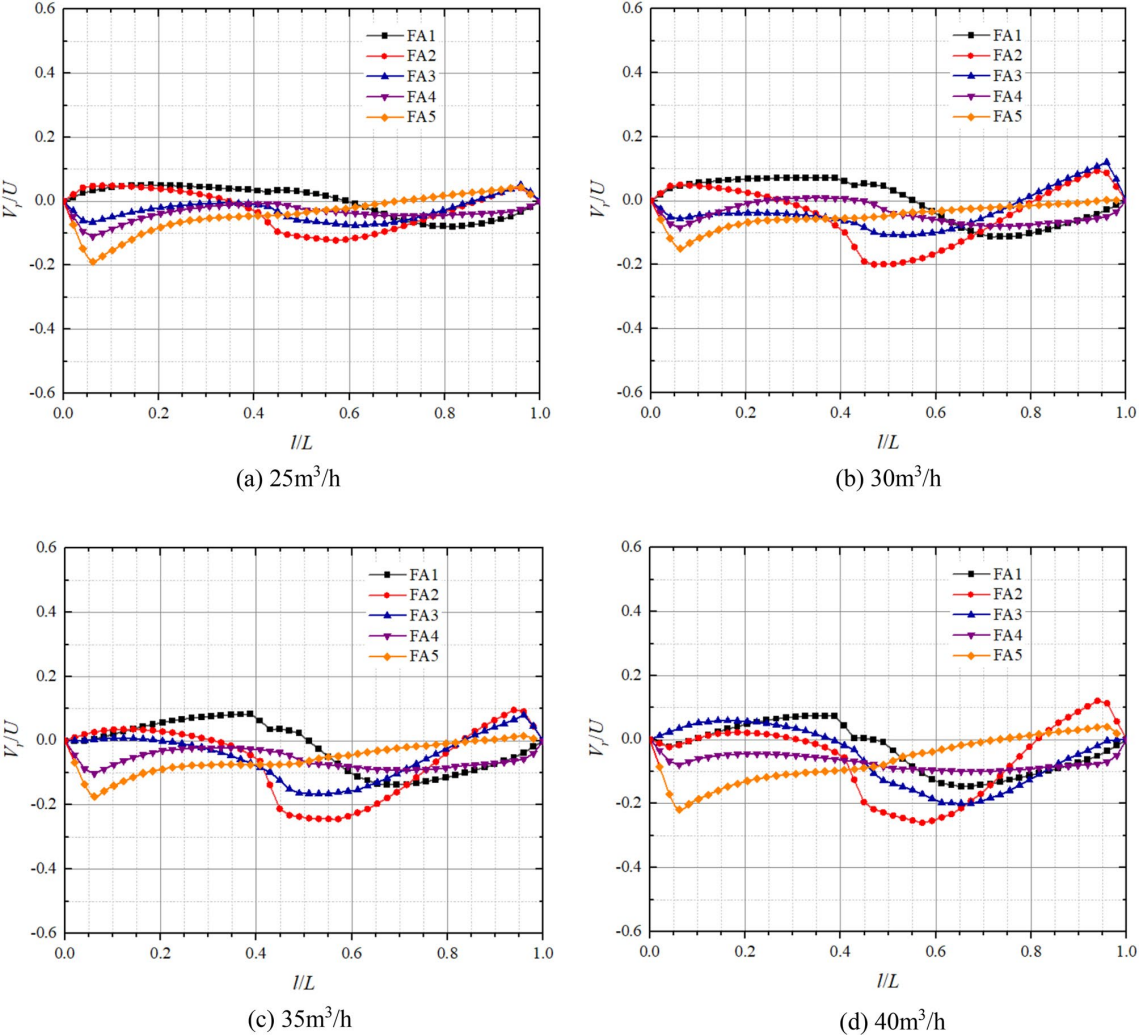
Dimensionless radial velocity axial distribution in the front chamber at turbine mode.

**Figure 16:**
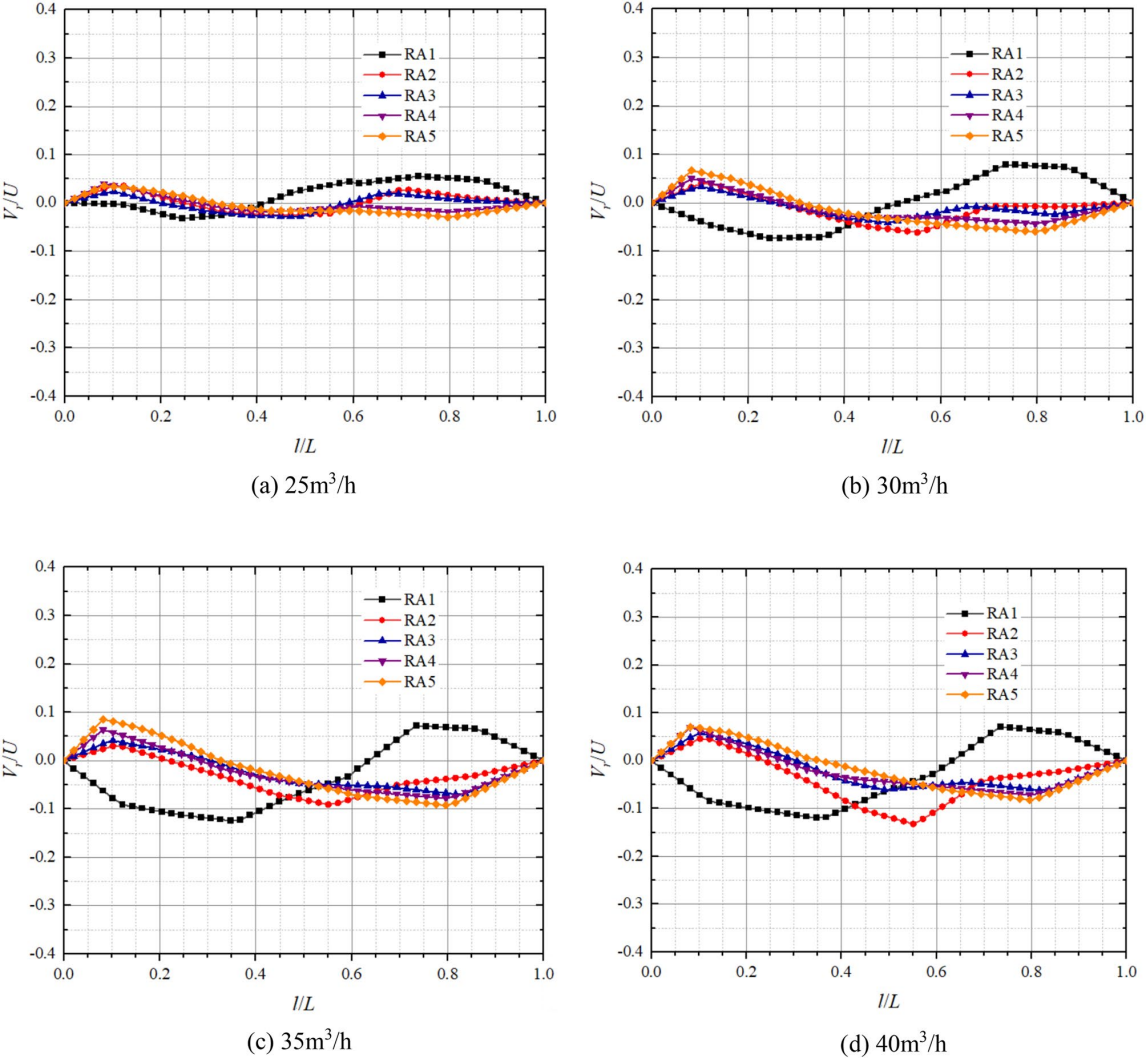
Dimensionless radial velocity axial distribution in the rear chamber at turbine mode.

The axial distribution of dimensionless radial velocities in the front and the rear chambers respectively in turbine mode is shown in Figs. 15 and 16. It can be found that the dimensionless radial velocities of the front and rear chambers in the turbine mode are small, with the dimensionless radial velocities mainly in the range of − 0.2 to 0.1 in the front chamber and − 0.1 to 0.1 in the rear chamber. Due to the presence of multiple vortices in the front chamber when the pump is working as turbine, the flow is extremely unstable, resulting in significant differences in the dimensionless radial velocity distribution compared to other operating conditions. The radial velocities on the front cover plate wall and pump case wall are all 0. FA4 and FA5 have a turbulent boundary layer at relative positions 0–0.06, where the centrifugal force has difficulty in overcoming the radial pressure difference and generates downward radial velocities, and the dimensionless radial velocities gradually decrease at 0.06–0.51, with smaller changes in dimensionless radial velocities at 0.51–1. FA1 and FA2 have turbulent boundary layers at 0.96–1. There is a significant dimensionless radial velocity variation at relative position 0.43, with varying degrees of velocity variation observed in both FA2 and FA3. At 25 m^3^/h, the dimensionless radial velocity of the rear chamber is much smaller, the radial velocity on both sides begins to slowly become larger as the flow gradually increases. It is worth noting that the dimensionless radial velocity at RA1 changes most obviously, and the direction of the velocity is the opposite of the other positions, which may be due to the existence of two small vortices at RA1, resulting in the opposite direction of the velocity here.

### Flow field analysis

**Figure 17:**
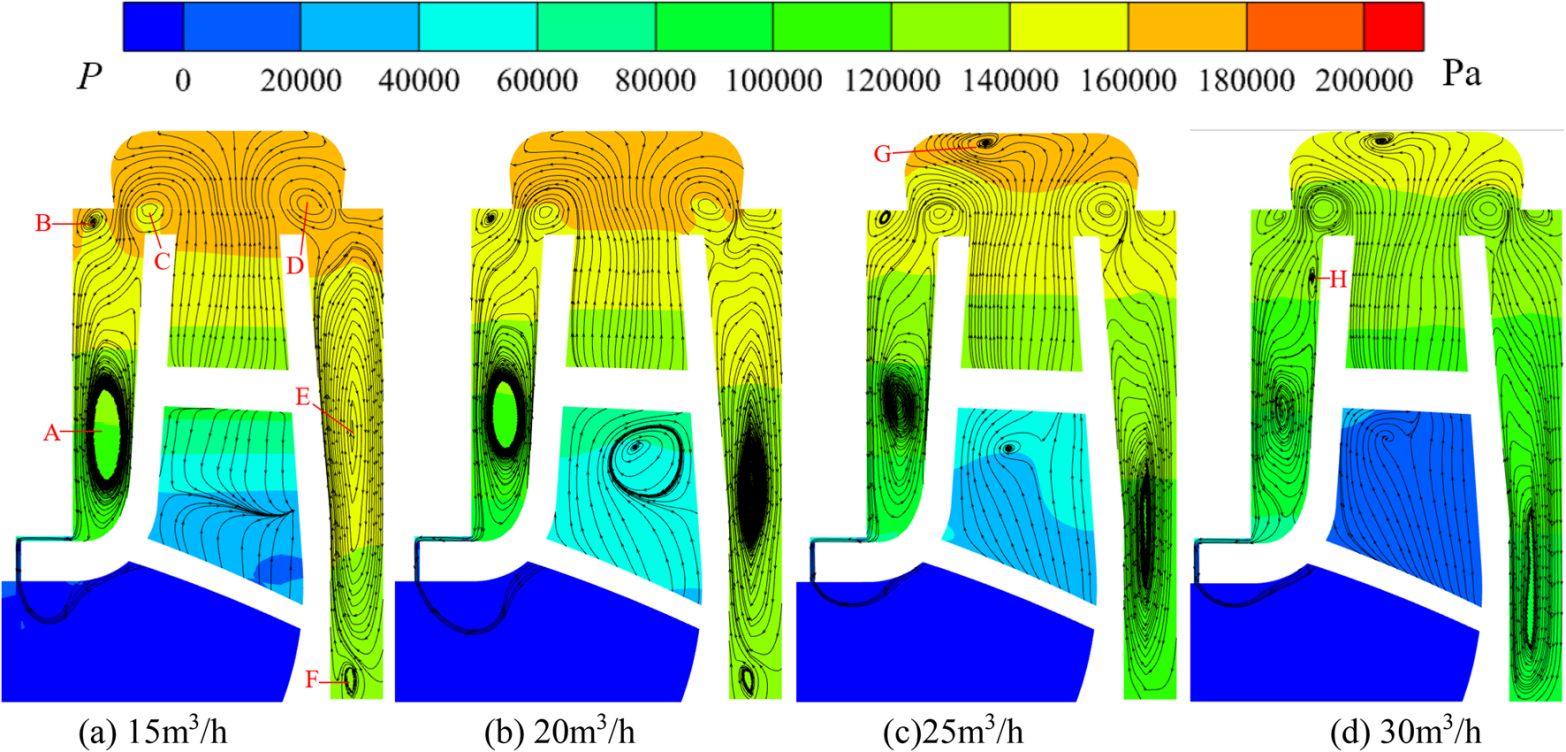
Pressure and streamline in the chamber at pump mode.

**Figure 18:**
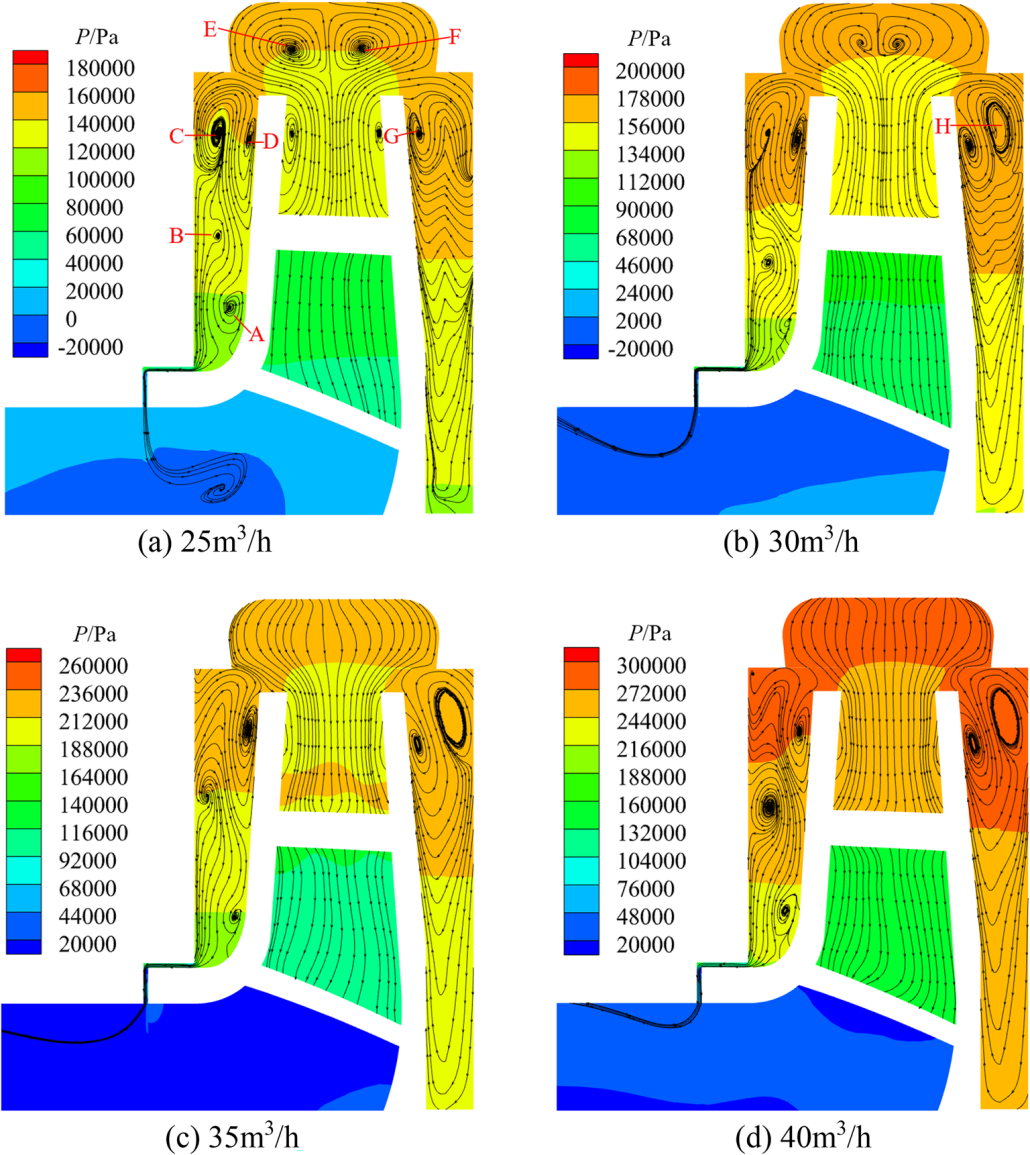
Pressure and streamline in the chamber at turbine mode.

Figures 17 and 18 show the pressure distribution and streamline of the front and rear chambers of the pump and turbine under different flows. The pressure variation pattern in the front and rear chambers of the pump and turbine is similar, and the pressure decreases in a stepped manner with the decrease of radius. At 25 m^3^/h, the pressure variation range in the front chamber of the pump is 80–140 kPa, and the range of pressure variation in the rear chamber is 107–140 kPa; at 35 m^3^/h, the pressure variation range in the front chamber of the turbine is 165–225 kPa, and the range of pressure variation in the rear chamber is 190–230 kPa. It can be concluded that the overall pressure inside the pump chamber in pump mode is lower than that in turbine mode under rated operating conditions.

At flows of 15 m^3^/h, 20 m^3^/h, 25 m^3^/h, 25 m^3^/h, the pressure variation range of the front chamber of the pump is 100–165 kPa, 87–157 kPa, 80–140 kPa, 73–132 kPa, and the pressure differences are 65 kPa, 70 kPa, 60 kPa, and 59 kPa, respectively; The pressure variation range of the rear chamber of the pump is 138–168 kPa, 122–160 kPa, 107–140 kPa, 93–133 kPa, and the pressure differences are 30 kPa, 38 kPa, 33 kPa, 40 kPa, respectively. At flows of 25 m^3^/h, 30 m^3^/h, 35 m^3^/h, 40m^3^/h, the pressure variation range of the front chamber of the turbine is 100–150 kPa, 126–174 kPa, 165–225 kPa, 214–284 kPa and the pressure differences are 50 kPa, 48 kPa, 60 kPa, 70 kPa, respectively; The pressure variation range of the rear chamber of the turbine is 119–152 kPa, 140–176 kPa, 190–230 kPa, 245–287 kPa and the pressure differences are 33 kPa, 36 kPa, 40 kPa, 42 kPa respectively. It can be found that the overall pressure inside the pump chamber in pump mode will gradually become smaller while the pressure variation pattern of turbine is the opposite with increasing flow. It can also be observed that the maximum pressure in the front and rear chambers under two modes is similar, but the pressure difference in front chamber is greater than that in the rear chamber. This is because the front chamber is connected to the pump inlet or the turbine outlet through the clearance of wear-rings, and the fluid in the front chamber can flow out from the clearance of wear-rings to release pressure, increasing the pressure difference in the front chamber.

At 15 m^3^/h, 20 m^3^/h, 25 m^3^/h, 25 m^3^/h for pump, the pressures at the clearance connected to the front chamber are about 30 kPa, 46 kPa, 43 kPa and 43 kPa respectively, showing a trend of increasing and then decreasing; The internal pressure variation range of the gap connected to the impeller inlet is about 6–8 kPa, 6–10 kPa, 8–10 kPa, and 10–13 kPa, respectively and the pressure increase with increasing flow and decreases with decreasing radius. At 25 m^3^/h, 30 m^3^/h, 35 m^3^/h, and 40 m^3^/h for turbine, the pressures at the clearance connected to the front chamber are about 48 kPa, 67 kPa, 103 kPa, and 128 kPa, respectively, showing a gradually increasing trend; The internal pressure variation range of the clearance connected to the impeller outlet is about 13–18 kPa, 8–13 kPa, 41–44 kPa, and 52–55 kPa, respectively. The internal pressure decreases with the decrease of radius and decreases first and then increases with increasing flow. The pressure at the turbine wear-ring clearance is significantly higher compared to the pump and pressure changes in the turbine wear-ring clearance are more susceptible to flow changes. In addition, the pressure at the junction of the front chamber and the clearance and at the corners of the clearance decreases suddenly and then rises gradually in both modes, with minimum pressures at the pressure drop of about 35 kPa and − 2.5 kPa for the pump and 75 kPa and 3.5 kPa for the turbine respectively at rated working conditions. In pump mode, the pressure distribution at the clearance between the volute and the impeller is uniform, and the pressure value is the same as the pressure at the maximum radius of the pump chamber; while the pressure distribution at the clearance in turbine mode shows a tendency to be higher on both sides and lower in the middle.

Figure 17 shows the axial streamlines of the pump, the flow circulates counterclockwise in the front chamber and circulates clockwise in the rear pump chamber under four working conditions. The circulation in the front chamber forms vortex A. The center position of vortex A will slowly move upwards with increasing flow. The radius of the center position of vortex A corresponding to the four operating conditions is 46.81 mm, 48.66 mm, 49.69 mm, and 49.72 mm, respectively; The center pressures of vortex A are 117 kPa, 114 kPa, 109 kPa, and 100 kPa, respectively. The flow inside the rear chamber forms vortices E and F. The center position of vortex E will gradually move downwards with increasing flow, while vortex F will gradually disappear. The center position radius of vortex E corresponding to the four operating conditions is 46.62 mm, 40.83 mm, 37.73 mm, and 32.42 mm, respectively; The center pressures of vortex E are 144 kPa, 130 kPa, 116 kPa, and 97 kPa, respectively. When the fluid flows into the chambers by reason of pressure difference from the impeller outlet, vortices C and D are formed, located respectively at the volute inlet. The change in flow has little effect on the positions of vortices C and D. The center pressures of vortex C are 156 kPa, 157 kPa, 148 kPa, and 131 kPa, respectively. The center pressures of vortex D are 164 kPa, 156 kPa, 147 kPa, and 133 kPa, respectively. Vortex B is located at the maximum radius of the front pump chamber and gradually disappears with increasing flow. At 30 m^3^/h, a vortex H was formed near the front cover plate wall r = 61.91 mm, with a central pressure of 123 kPa.

From the axial streamlines of the turbine shown in Fig. 18, it can be seen that compared to centrifugal pump, the flow inside the chambers is more complex when the pump works as a turbine. As the fluid flows towards the impeller inlet from the pump chamber, a portion of it flows back into the front chamber, forming vortices C and D. The flow inside the chambers will increases with increasing system flow, and vortex C will gradually move downwards. The corresponding position radius of vortex C under the four flow conditions is 61.87 mm, 61.84 mm, 54.84 mm, and 53.45 mm, respectively; The center pressures of vortex C are 138 kPa, 171 kPa, 210 kPa, and 256 kPa, respectively. The change in flow has little effect on the position of vortex D, which is located near r = 61 mm; The center pressures of vortex D are 140 kPa, 168 kPa, 220 kPa, and 273 kPa, respectively. A portion of the fluid in the front chamber will flow into the outlet of the turbine from the clearance of wear-rings, forming vortices A and B during the flow towards the clearance. Vortex A is located near r = 43 mm, and its central position is not significantly affected by changes in flow; The center pressures of vortex A under four operating conditions are 117 kPa, 139 kPa, 186 kPa, and 235 kPa, respectively. But as the flow increases, vortex B gradually disappears. At 25 m^3^/h and 30 m^3^/h, there are vortices E and F in the volute, which disappear with increasing flow, indicating that the flow in the volute will gradually stabilize with increasing flow during turbine operation. During the flow of fluid towards the impeller inlet in the rear chamber, a portion of the fluid flows back to form vortices G and H. The change in flow has little effect on the center position of vortices G and H, which are located at r = 60 mm and r = 63 mm, respectively. The center pressures of vortex G are 149 kPa, 172 kPa, 227 kPa, and 281 kPa, respectively; the center pressures of vortex H are 174 kPa, 229 kPa, and 281 kPa, respectively. After reaching the bottom wall, the fluid in the rear chamber will continue to participate in vortex circulation along the wall of the rear cover plate.

### Stability analysis

In this paper, the energy gradient method is used to process the calculated flow field data, and the instability inside the pump is analyzed by obtaining the distribution of energy gradient function in the flow channel. As of now, the energy gradient theory has been successfully applied in many fluid flow fields^30–32^, and the numerical simulation results based on the energy gradient theory are basically in agreement with previous research results and experimental results. These agreements confirm that the theory can reveal the physical mechanism of fluid flow instability mentioned above.

The energy gradient parameter of centrifugal pump is calculated as^33^:

5 $$ \begin{gathered} K = \frac{\partial E}{{\partial {\text{n}}}}\left( {\frac{\partial H}{{\partial s}}} \right)^{ - 1} = \frac{{\frac{\partial p}{{\partial n}} + \rho U\frac{\partial U}{{\partial n}}}}{{\frac{{\mu_{t} }}{U}\left( {\frac{\partial U}{{\partial n}}} \right)^{2} - \frac{{2\mu_{t} }}{{\rho U^{2} }}\frac{\partial U}{{\partial n}}\frac{\partial p}{{\partial n}} + \frac{{\mu_{t} }}{{\rho^{2} U^{3} }}\left( {\frac{\partial p}{{\partial n}}} \right)^{2} }} \\ \\ \end{gathered} $$

6 $$ E = p + \frac{1}{2}\rho \left( {u^{2} + v^{2} + w^{2} } \right) $$

where $p$—static pressure; $u$—x-axis velocity component; $v$—y-axis velocity component; $w$—z-axis velocity component; ${\text{n}}$—normal direction of fluid flow; $s$—streamline direction of fluid flow; $H$—mechanical energy lost in the flow; $\rho$—fluid density; $E$—total pressure; $U = \left( {u^{2} + v^{2} + w^{2} } \right)^{\frac{1}{2}}$.

**Figure 19:**
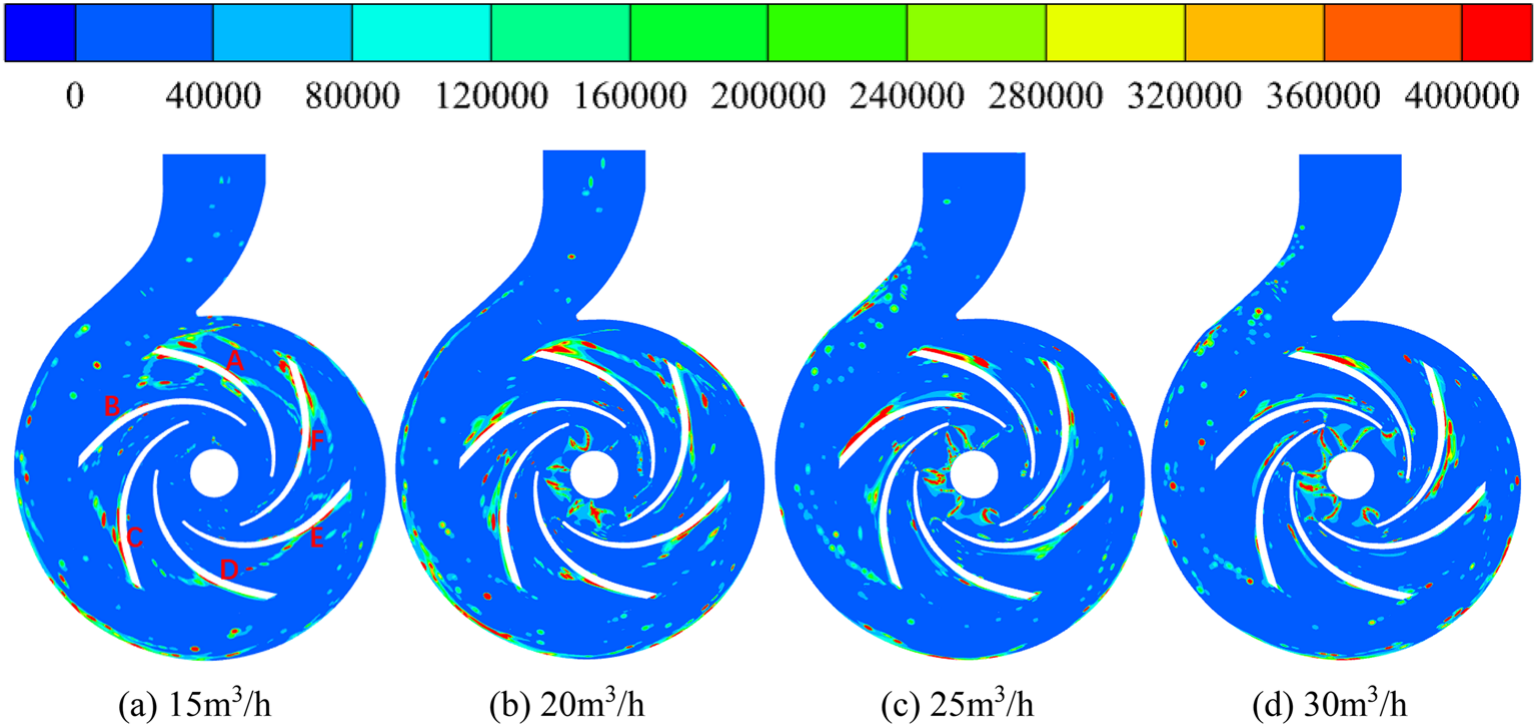
Distribution of energy gradient function K at pump mode.

The distribution of K in the internal fluid domain of the centrifugal pump at different flows is obtained by numerical calculations, as shown in Fig. 19. The red area is a high K value area, where the higher the K value, the stronger the turbulence intensity, and the more unstable the flow. In the four operating conditions, the areas with high K values are mainly distributed near the blades working surface near the impeller outlet, indicating that instability first occurs from the impeller outlet and working surface side with increasing flow. The unique structure of the volute tongue results in a large area of high K value near the working face of blade A. At the flows of 15 m^3^/h, 20 m^3^/h, 25 m^3^/h and 30 m^3^/h, the K value near the working surface of blade A varies in the ranges of 50,000–390,000, 70,000–550,000, 90,000–590,000 and 12,000–63,0000, respectively. It follows that the area of the region with larger K values gradually increases and changes from a block to a band with increasing flow, and red area will also gradually increase in size. At 25 m^3^/h, the working surface of blade B shows a large area of K. Its red area is larger than that of blade A, and the center K value reaches a maximum of 800,000. The high K value area near blade B decreases and disappears with increasing flow. There are also large high K value areas on the working surface of blade F, but the red area is significantly smaller than that of blade A. When the flows are 15 m^3^/h, 20 m^3^/h, 25 m^3^/h, and 30 m^3^/h, the K values in most areas are between 110,000 and 240,000. The reason why the K value area is mainly distributed near the impeller outlet is that in the impeller outlet and volute, the flow has unstable periodicity and confirmed circumferential distortion in the pressure distribution. The pressure fluctuation caused by the interaction between the impeller flow channel and the volute basin is most intense at the impeller outlet^34^. At 20 m^3^/h, 25 m^3^/h and 30 m^3^/h, there is also a scattered distribution of large K values at impeller inlet. The area with K values ranging from 160,000 to 180,000 will gradually decrease with increasing flow. The K value near the volute inner wall is also large, and its area will decrease with increasing flow. This is due to the influence of jet wake, which leads to unstable flow in the diffuser channel.

### Entropy generation analysis

The entropy generation theory and energy gradient method are both based on vortex dynamics to amplify local poor flow inside the pump and study their relationship with hydraulic performance. The entropy production theory is an effective tool to intuitively reflect the location of irreversible losses^35^ within the fluid and the spatial distribution of energy consumption.

For turbulence, entropy production has two parts: one part is caused by time-averaged motion; The other part is caused by transient speed fluctuations. Therefore, Ṡ‴ (Entropy Production Rate, EPR) can be expressed using the following equation^36^:

7 $$ {{\dot{S}^{\prime\prime\prime} = \dot{S}^{\prime\prime\prime}}}_{D} + {{\dot{S}^{\prime\prime\prime\prime}}}_{D} $$

The entropy production caused by time averaged and pulsation is shown in Eqs. (3) and (4):

8 $$ \dot{S}^{\prime\prime\prime}_{D} = \frac{2\mu }{T}\left[ {\left( {\frac{{\partial \overline{u}}}{\partial x}} \right)^{2} + \left( {\frac{{\partial \overline{v}}}{\partial y}} \right)^{2} + \left( {\frac{{\partial \overline{w}}}{\partial z}} \right)^{2} } \right] + \frac{\mu }{T}\left[ {\left( {\frac{{\partial \overline{v}}}{\partial x} + \frac{{\partial \overline{u}}}{\partial y}} \right)^{2} + \left( {\frac{{\partial \overline{w}}}{\partial x} + \frac{{\partial \overline{u}}}{\partial z}} \right)^{2} + \left( {\frac{{\partial \overline{v}}}{\partial z} + \frac{{\partial \overline{w}}}{\partial y}} \right)^{2} } \right] $$

9 $$ \dot{S}^{\prime\prime}_{{D^{\prime}}} = \frac{{2\mu_{eff} }}{T}\left[ {\left( {\frac{{\partial u^{\prime}}}{\partial x}} \right)^{2} + \left( {\frac{{\partial v^{\prime}}}{\partial y}} \right)^{2} + \left( {\frac{{\partial w^{\prime}}}{\partial z}} \right)^{2} } \right] + \frac{{\mu_{eff} }}{T}\left[ {\left( {\frac{{\partial v^{\prime}}}{\partial x} + \frac{{\partial u^{\prime}}}{\partial y}} \right)^{2} + \left( {\frac{{\partial w^{\prime}}}{\partial x} + \frac{{\partial u^{\prime}}}{\partial z}} \right)^{2} + \left( {\frac{{\partial v^{\prime}}}{\partial z} + \frac{{\partial w^{\prime}}}{\partial y}} \right)^{2} } \right] $$

where: $\dot{S}^{\prime \prime \prime }_{D}$—average entropy production rate of velocity; $\dot{S}_{{D^{\prime } }}^{\prime \prime }$—entropy production of velocity pulsation; *μ*—kinematic viscosity; $\overline{u}$, $\overline{v}$, $\overline{w}$—time averaged velocities; $u_{{}}^{\prime }$, $v_{{}}^{\prime }$,$w_{{}}^{\prime }$—pulsating velocities; *T*—temperature, set the temperature to a constant of 293 K during calculation; $\mu_{eff}$—effective kinematic viscosity, as shown in Eq. (10):

10 $$ \mu_{eff} = \mu + \mu_{t} $$

$\mu_{t}$is the viscosity of turbulent motion.

$\dot{S}^{\prime\prime\prime}_{D}$ can be solved directly by numerical computation, while $\dot{S}_{{D^{\prime } }}^{\prime \prime }$ cannot be solved directly by numerical computation. According to the local entropy production theory of Kock^37^, the entropy production caused by velocity fluctuation is related to ε or ω of the turbulence model. Therefore, the expression for local entropy production caused by velocity fluctuations^38^ is:

11 $$ \dot{S}_{{D^{\prime\prime\prime}}} = \alpha \frac{\rho \omega k}{T} $$

$\alpha$ = 0.09, $\omega$— turbulent vortex frequency, /s; *k*—turbulence intensity, m^2^/s^2^.

Due to the strong wall effect and more pronounced time-averaged term in the entropy production rate, the entropy production near the wall is calculated as:

12 $$ S_{pro,W} = \int_{S}^{{}} {\frac{{\vec{\tau }.\vec{\nu }}}{T}} dS $$

$\tau$—wall shear stress, Pa; *S*—area, m^2^; $v$—near wall velocity, m/s.

Therefore, the total entropy production in the computational domain of the whole system is calculated as:

13 $$ S_{{{pro}}} = \int_{V}^{{}} {\dot{S}^{\prime\prime\prime}_{D} } dV + \int_{V}^{{}} {\dot{S}^{\prime\prime\prime}_{{D^{\prime}}} } dV + \int_{S}^{{}} {\frac{{\vec{\tau }.\vec{\nu }}}{T}} dS $$

**Figure 20:**
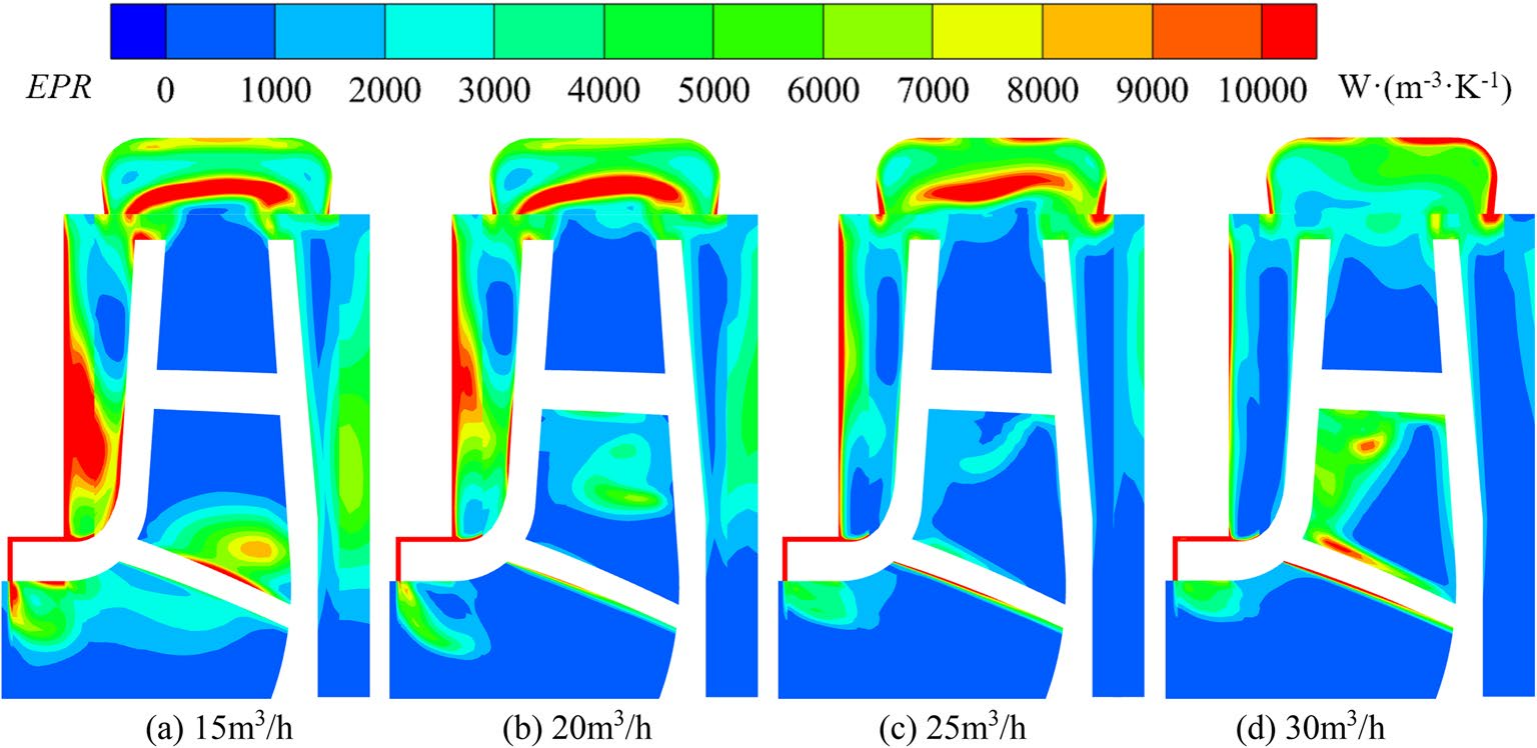
Entropy production distribution at pump mode.

The local entropy production distribution cloud map of the chambers under different flows in pump mode is shown in Fig. 20. The high entropy production area in the pump chamber gradually decreases with increasing flow, indicating that the energy loss inside the pump chambers gradually decreases with increasing flow. The high entropy production area and the maximum value in the front chamber are larger than those in rear chamber. At 15 m^3^/h, the high entropy production area is the largest and is mainly concentrated in the range of r = 40.65 mm to r = 56.50 mm in the front chamber, which extends from the side of the pump casing to the front cover plate side, and the maximum value reaches 21,597 W/(m^3^ K); the overall entropy production in the rear chamber is relatively low, with a relatively high entropy production in the range of r = 33.65 mm to r = 56.09 mm, but its maximum value is only 6670 W/(m^3^ K). At 20 m^3^/h, the range of high entropy production in the front pump chamber decreases to r = 46.66 mm ~ r = 57.61 mm, with a maximum value of 10,344 W/(m^3^ K); the range of entropy production area in the rear pump chamber is further reduced. The red area in the front pump chamber gradually disappears with increasing flow, and the entropy production decreases along the pump casing wall towards the inside of the pump chamber. Due to the strong wall effect, it can be found that the fluid domain in the front chamber near the pump wall and the clearance of wear-ring has been in high entropy production state, and the value has been higher than 10,000 W/(m^3^ K), which indicates that the energy loss near the wall is high. At 15 m^3^/h, a high entropy production area is also formed near the front cover plate wall at r = 37.80 mm to r = 50.20 mm. As the flow increased, its area and maximum value both decreased. In summary, the energy loss in front pump chamber is greater and the unstable flow in the chambers at low flows also causes significant energy loss.

**Figure 21:**
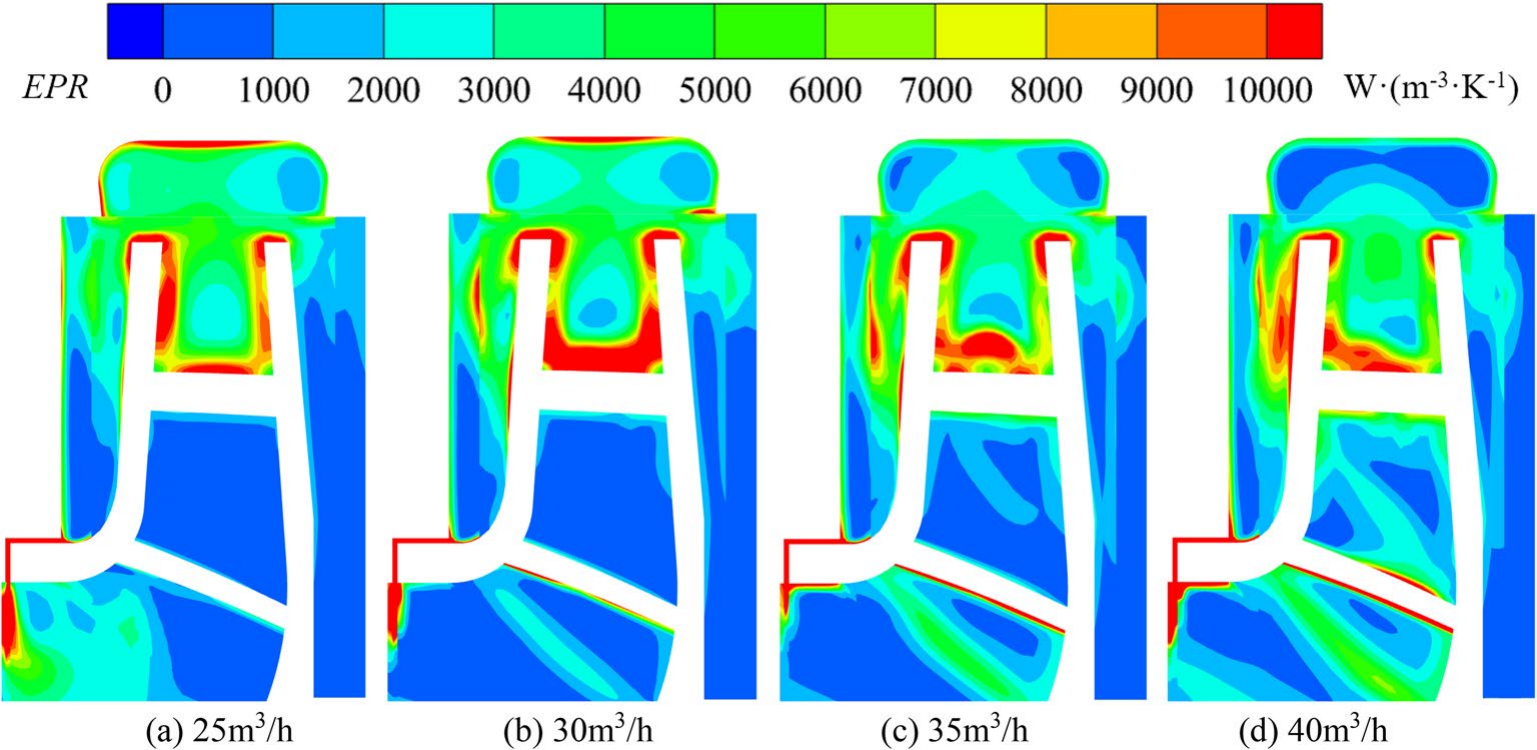
Entropy production distribution at turbine mode.

The local entropy production distribution cloud map of the chambers under different flows in turbine mode is shown in Fig. 21. The entropy production change inside the front and rear chambers during turbine operation is opposite to that of the pump. The internal entropy production value and area gradually increase with increasing flow. The high entropy production area in the front chamber is concentrated within the range of r = 47.08 mm to r = 66.08 mm, extending from the front cover plate wall towards the interior of the chamber. At 25 m^3^/h, two high entropy production areas were formed on the front cover plate wall from r = 49.66 mm to r = 55.31 mm and from r = 61.47 mm to r = 66.82 mm and the area gradually increased and connected into one. At 30 m^3^/h, a striped high entropy production area appears in the middle of the front pump chamber from r = 55.85 mm to r = 63.83 mm, with a center entropy production value of 10,669 W/(m^3^ K); at 35 m^3^/h, its position moves down to the range of r = 52.73 mm to r = 63.06 mm, and the central value is 11,417 W/(m^3^ K). It gradually expands to the wall surface of front cover plate with increasing flow; At 40 m^3^/h, the maximum value at the center is 10,320 W/(m^3^ K). The entropy production distribution near the pump casing wall in the front chamber is uniform, with values ranging from 4000 to 6000 W/(m^3^ K). The entropy production value of the rear chamber has always been in a low state, gradually decreasing from the junction of the rear chamber and the volute towards the interior of the chamber. Similar to the pump, the entropy production value at the clearance of wear-ring during turbine operation has always been in a high state, with an average value higher than 10,000 W/(m^3^ K), which indicates that clearance of wear-ring has been a region with high energy loss.

### Internal pressure difference analysis

**Figure 22:**
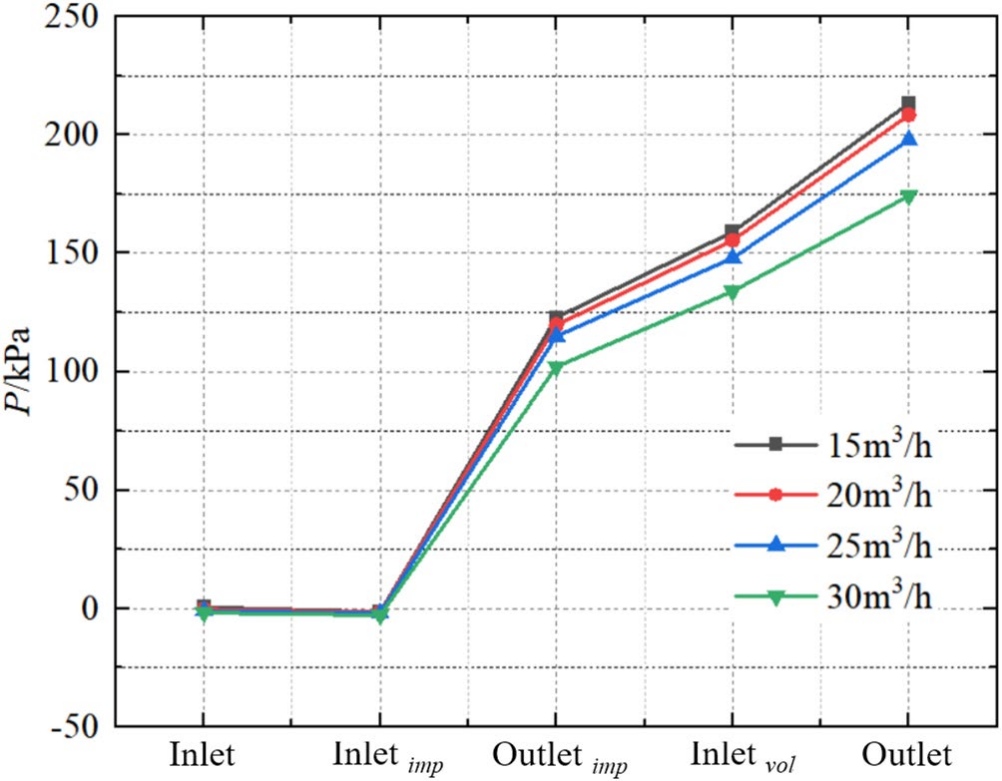
Internal pressure change at pump mode.

The internal pressure change in pump mode is shown in Fig. 22. The pressure on all five monitoring surfaces all decreases with increasing flow. The inlet pressures at flows of 15 m^3^/h, 20 m^3^/h, 25 m^3^/h, and 30 m^3^/h are 0.489 kPa, − 0.332 kPa, − 1.035 kPa, and − 1.865 kPa, respectively. During the process of fluid flowing from the extended section of the pump inlet to the impeller inlet, the pressure slightly decreases. The corresponding pressures on the impeller inlet surface from small to large flows are − 1.463 kPa, − 1.598 kPa, − 1.997 kPa, and − 2.924 kPa, respectively; the pressure differences are − 1.952 kPa, − 1.266 kPa, − 0.962 kPa, and − 1059 kPa, respectively. When the fluid passes through the impeller, the kinetic energy of the impeller is converted into the pressure energy of the fluid, and the fluid pressure rises sharply. The pressures on the outlet surface of the impeller corresponding to the flow from small to large are 122.871 kPa, 119.726 kPa, 114.969 kPa, and 102.062 kPa, respectively; The pressure differences are 124.334 kPa, 121.324 kPa, 116.966 kPa, and 104.986 kPa, respectively. The pressures at the inlet surface of the volute corresponding to the flow from small to large are 158.961 kPa, 155.416 kPa, 148.07 kPa, and 133.993 kPa, respectively; The pressure differences from the impeller outlet to the volute inlet are 36.09 kPa, 35.69 kPa, 33.101 kPa, and 31.931 kPa, respectively. The pressure further increases when the fluid passes through the expansion chamber and extends to the pump outlet. The pressures on the pump outlet surface corresponding to the flow from small to large are 213.383 kPa, 208.609 kPa, 197.965 kPa, and 174.286 kPa, respectively, with a pressure ratio of 1.22:1.20:1.14:1; The pressure differences from the volute inlet to the volute outlet are 54.422 kPa, 53.193 kPa, 49.895 kPa, and 40.293 kPa, respectively. In summary, it can be found that the pressure difference between the monitoring surfaces decreases with increasing flow, but the pressure difference between the centrifugal pump inlet and the impeller inlet shows a tendency to first decrease and then increase.

**Figure 23:**
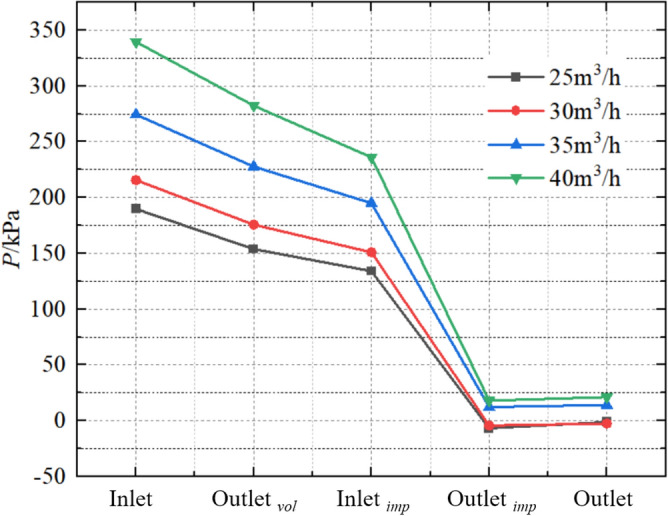
Internal pressure change at turbine mode.

The internal pressure change in turbine mode is shown in Fig. 23. The pressure on all five monitoring surfaces shows an increasing trend with increasing flow. At 25 m^3^/h, 30 m^3^/h, 35 m^3^/h and 40 m^3^/h, the corresponding inlet pressures are 189.745 kPa, 215.685 kPa, 274.313 kPa and 339.623 kPa, respectively. When the fluid flows to the outlet of the volute, the pressure under each operating condition decreases to 153.853 kPa, 175.752 kPa, 227.854 kPa, and 282.397 kPa, respectively; the pressure difference are − 35.892 kPa, − 39.933 kPa, − 46.459 kPa, and − 57.626 kPa, respectively. The inlet pressures of the impeller corresponding to the flow from small to large are 134.022 kPa, 150.969 kPa, 194.92 kPa, and 236.062 kPa, respectively; The pressure differences with the outlet s of volute are − 19.831 kPa, − 24.783 kPa, − 32.934 kPa, and − 46.335 kPa, respectively. After the fluid flows into the impeller channel, the pressure energy of the fluid is converted into the rotational mechanical energy of the shaft system, and the pressure of the fluid drops sharply. The corresponding outlet pressures of the impeller corresponding to the flow from small to large are − 6.849 kPa, − 4.444 kPa, 12.209 kPa, and 17.947 kPa, respectively; The pressure differences are − 140.871 kPa, − 155.413 kPa, − 182.711 kPa, and − 218.115 kPa, respectively. When the fluid flows to the extended section of the system outlet, the pressure slightly increases. The corresponding system outlet pressures for the flow from small to large are − 1.258 kPa, − 2.622 kPa, 13.933 kPa, and 21.039 kPa, respectively; the outlet pressure ratio is (-1): (-2.08): (11.08): (16.72); The pressure differences are 5.591 kPa, 1.822 kPa, 1.724 kPa, and 3.092 kPa, respectively. In summary, the pressure difference between the monitoring surfaces increase with increasing flow.

## Conclusion

1. The leakage flow from the clearance of wear-ring of the centrifugal pump will gradually decrease with increasing flow, and the volumetric efficiency will increase rapidly; The turbine volumetric efficiency is less affected by the flow and slowly increases with increasing flow.
2. The distribution patterns of dimensionless circumferential velocity and dimensionless radial velocity in the front and rear chambers of centrifugal pump and pump as turbine have similarities. The flow inside the pump chamber is divided into turbulent boundary layer and core flow zone. The dimensionless circumferential velocity on the cover plate side is much greater than that on the pump wall side, and the radial difference of the dimensionless circumferential velocity in the core flow area in the front chamber is more significant than that in the rear chamber under both operating modes. The maximum dimensionless radial velocity in the pump mode occurs at the junction of the turbulent boundary layer and the core flow zone, and the maximum dimensionless radial velocity on the pump casing side is greater than that on the cover plate side. The overall dimensionless radial velocity in the turbine mode is relatively small. The radial velocity direction at the maximum radius of the rear chamber is opposite to other positions.
3. The flow instability phenomenon first appears on the blade working surface and impeller outlet with increasing flow; the flow instability phenomenon at the impeller inlet gradually disappear as the flow rate decreases.
4. The high entropy production area in the front chamber of the two operating modes is always larger than that in the rear chamber. The high entropy production area in the chambers decreases with increasing flow in pump mode, while the opposite is true for turbine. The clearance of wear-ring is the place with the most energy loss.
5. The pressure inside the front and rear chambers of the pump and turbine shows a gradient distribution, which decreases with the decrease of radius. The pressure difference in the front chamber is greater than that in the rear chamber. In pump mode, the internal pressure of the pump chamber increases with the increase of flow, while in turbine mode, the opposite is true.

## Data Availability

The data that support the findings of this study are available from the corresponding author upon reasonable request.
